# Competition between One‐ and Two‐Electron Unimolecular Reactions of Late 3d‐Metal Complexes [(Me_3_SiCH_2_)*_n_* M]^–^(M = Fe, Co, Ni, and Cu; *n* = 2–4)

**DOI:** 10.1002/anie.202500524

**Published:** 2025-04-04

**Authors:** Torben Kühl, Lisa Hetzel, Christopher J. Stein, Konrad Koszinowski

**Affiliations:** ^1^ Institut für Organische und Biomolekulare Chemie Universität Göttingen Tammannstraße 2 Göttingen 37077 Germany; ^2^ TUM School of Natural Sciences and Catalysis Research Center Department of Chemistry Technical University of Munich Lichtenbergstraße 4 Garching 85748 Germany; ^3^ Atomistic Modeling Center Munich Data Science Institute Technical University of Munich Garching 85748 Germany; ^4^ Friedrich‐Wöhler‐Forschungsinstitut für Nachhaltige Chemie Universität Göttingen Tammannstraße 2 Göttingen 37077 Germany

**Keywords:** 3d Metals, Gas‐phase reactions, Quantum chemical calculations, Radical reactions, Reductive eliminations

## Abstract

Although organometallic complexes of the late 3d elements are known to undergo both one‐and two‐electron reactions, their relative propensities to do so remain poorly understood. To gain direct insight into the competition between these different pathways, we have analyzed the unimolecular gas‐phase reactivity of a series of well‐defined model complexes [(Me_3_SiCH_2_)*_n_* M]^−^ (M = Fe, Co, Ni, and Cu; *n* = 2–4). Applying a combination of tandem‐mass spectrometry, quantum‐chemical computations, and statistical rate‐theory calculations, we find several different fragmentation reactions, among which the homolytic cleavage of metal‐carbon bonds and radical dissociations are particularly prominent. In all cases, these one‐electron reactions are entropically favored. For the ferrate and cobaltate complexes, they are also energetically preferred, which explains their predominance in the corresponding fragmentation experiments. For [(Me_3_SiCH_2_)_4_Ni]^−^ and, even more so, for [(Me_3_SiCH_2_)_4_Cu]^−^, a concerted reductive elimination as a prototypical two‐electron reaction is energetically more favorable and gains in importance. [(Me_3_SiCH_2_)_3_Ni]^−^ is special in that it has two nearly degenerate spin states, both of which react in different ways. A simple thermochemical analysis shows that the relative order of the first and second bond‐dissociation energies is of key importance in controlling the competition between radical dissociations and concerted reductive eliminations.

## Graphical abstract

Gas‐phase fragmentation experiments and theoretical calculations on a series of model complexes determine the intrinsic tendencies of the late 3d metals to undergo one‐versus two‐electron reactions and unravel the factors controlling the selectivity.

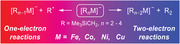

## Introduction

Transition metals are distinguished by an exceptionally versatile reactivity, for which reason they are indispensable to catalysis. Traditionally, the late 4d and 5d metals ruthenium, rhodium, palladium, iridium, and platinum have been considered the most important and used in countless applications.^[^ ^1^ ^]^ However, these precious metals not only are costly, but typically also require fine‐tuned and expensive ligands. Hence, reactions catalyzed by these metals do not score well in terms of sustainability, which becomes more and more important.^[^ ^2^ ^]^ In this respect, the late 3d metals iron, cobalt, nickel, and copper form a promising alternative and are increasingly applied.^[^ ^3^, ^4^, ^5^, ^6^, ^7^, ^8^, ^9^, ^10^, ^11^ ^]^ They are much cheaper than their heavier homologues and in many cases show the desired reactivity without the need for complex and expensive ligands. However, the 3d metals differ from most of their heavier homologues and, in particular, from palladium, in that they not only undergo two‐electron processes, such as concerted reductive eliminations, but have a higher tendency toward one‐electron reactions, such as homolytic bond cleavages.^[^ ^3^, ^12^, ^13^, ^14^, ^15^ ^]^

So far, the development of effective 3d‐metal catalysts often remains difficult and time consuming. The chief reason for this shortcoming lies in our limited knowledge of their reactivity.^[^ ^16^, ^17^ ^]^ To date, the reactions of organoiron, ‐cobalt, ‐nickel, and ‐copper species are not as well understood as those of organopalladium species.^[^ ^18^ ^]^ Thus, trends in the reactivity of the different 3d metals remain obscure. First, this poor understanding reflects the lower stability of organometallic complexes derived from the 3d elements.^[^ ^16^, ^17^ ^]^ These species, therefore, easily elude a systematic mechanistic analysis. Second, many 3d‐metal complexes tend to undergo redox‐disproportionation reactions in solution. The resulting simultaneous presence of complexes in different oxidation states gives rise to ill‐defined sample solutions and severely complicates mechanistic analyses.^[^ ^19^ ^]^ In particular, it renders it practically impossible to infer the microscopic reactivity of the individual species.

One approach to overcome this fundamental difficulty relies on gas‐phase studies of isolated ionic complexes. Tandem‐mass spectrometry permits the mass selection of the ions of interest and the preparation of well‐defined reactants, whose microscopic reactivity can then be probed under the rigorous exclusion of bimolecular exchange processes.^[^ ^20^, ^21^, ^22^, ^23^, ^24^ ^]^ The absence of counter‐ions and solvent molecules eliminates further interfering effects and thereby makes a comparison with the predictions of quantum‐chemical calculations straightforward. These calculations provide structural and energetic information, which the mass‐spectrometric experiments alone do not afford. The latter, in turn, can serve as a benchmark for testing the accuracy of the theoretical calculations.

**Scheme 1:**
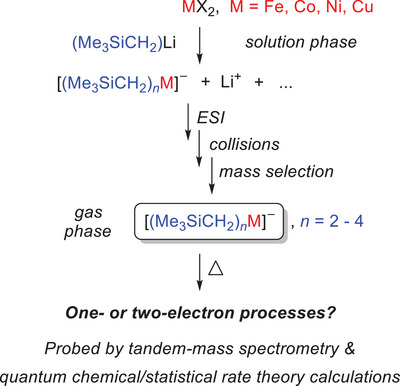
Design and scope of the present study.

This approach has proven well‐suited for determining the intrinsic reactivity of transition‐metal complexes and has been applied to numerous individual systems.^[^ ^25^, ^26^, ^27^, ^28^, ^29^ ^]^ However, as most of these individual systems differ in the number and nature of the organyl groups bound to the metal, no direct comparison between the different studies is feasible, thus preventing a systematic analysis of trends in reactivity. In the present work, we seek to accomplish such a systematic analysis by probing a series of [(Me_3_SiCH_2_)*_n_* M]^−^ model complexes (M = Fe, Co, Ni, and Cu; *n* = 2–4). By keeping the nature of the metal‐bound organyl group constant, we can directly compare the influence of the metal and its oxidation state on the reactivity. For assessing as many complexes as possible, the choice of the organyl group is of crucial importance. Organyl groups with β‐hydrogen atoms are to be avoided due to their propensity toward β‐hydrogen eliminations.^[^ ^1^ ^]^ Exploratory experiments with methyl‐ and phenyl‐containing complexes furthermore showed that only a limited number of these species were accessible. Instead, we found that the introduction of the Me_3_SiCH_2_ group permitted the preparation of nearly all of the sought‐after [(Me_3_SiCH_2_)*_n_* M]^−^ complexes, *n* = 2–4, and their transfer into the gas phase via electrospray ionization (ESI). The mass‐selected complexes of interest were then subjected to fragmentation reactions at different collision energies (Scheme 1). In parallel, we probed the unimolecular reactivity of the [(Me_3_SiCH_2_)*_n_* M]^−^ ions by quantum‐chemical calculations. On the basis of the computed potential energy surfaces, we performed statistical rate‐theory calculations to obtain rate constants of the different fragmentation reactions, thus allowing a direct comparison with the experimental observations.^[^ ^30^, ^31^ ^]^ Together, experiment and theory give detailed insight into the intrinsic reactivity of late 3d‐metal complexes and their tendency to undergo one‐ or two‐electron processes. When extrapolating the reactivity trends derived from the gas‐phase model systems to the situation in solution, due care must be taken to account for the effect of solvation.

## Results and Discussion

### Preparation of Organometalates [R*_n_* M]^−^

Gas‐phase organometalates of iron, cobalt, nickel, and copper were generated via negative‐ion mode ESI of reaction mixtures of the corresponding transition‐metal precursor and (trimethylsilyl)methyl lithium (RLi) in tetrahydrofuran at low temperatures (for full reaction details, see Table S1 in the Supporting Information). Besides transmetalation reactions, redox‐disproportionation processes and possibly also reductive eliminations are expected to proceed in solution,^[^ ^32^, ^33^, ^34^, ^35^, ^36^ ^]^ thereby increasing the number of accessible organometalate anions.^[^ ^37^ ^]^ Likewise, energetic collisions of the ions with neutral molecules during the ESI process and the subsequent ion‐transfer stage of the used quadrupole time‐of‐flight hybrid instrument (for the applied instrumental settings, see Table S2) supposedly result in further species. Accordingly, we succeeded in the preparation of all of the mononuclear homoleptic [R*_n_* M]^−^ complexes, *n* = 2–4, except for [R_3_Cu]^−^ (Figures S1–S6; for a list of the observed ions, see Table S3). In addition, the obtained ESI‐mass spectra also pointed to the presence of polynuclear organometalates as well as ions originating from ligand fragmentation and oxidation or hydrolysis reactions. These species were of no interest in the present context, but did not pose any problems because they were easily removed by the mass‐selection step in the spectrometer.

### Electronic Ground States of Organometalates [R*_n_* M]^−^

**Figure 1:**
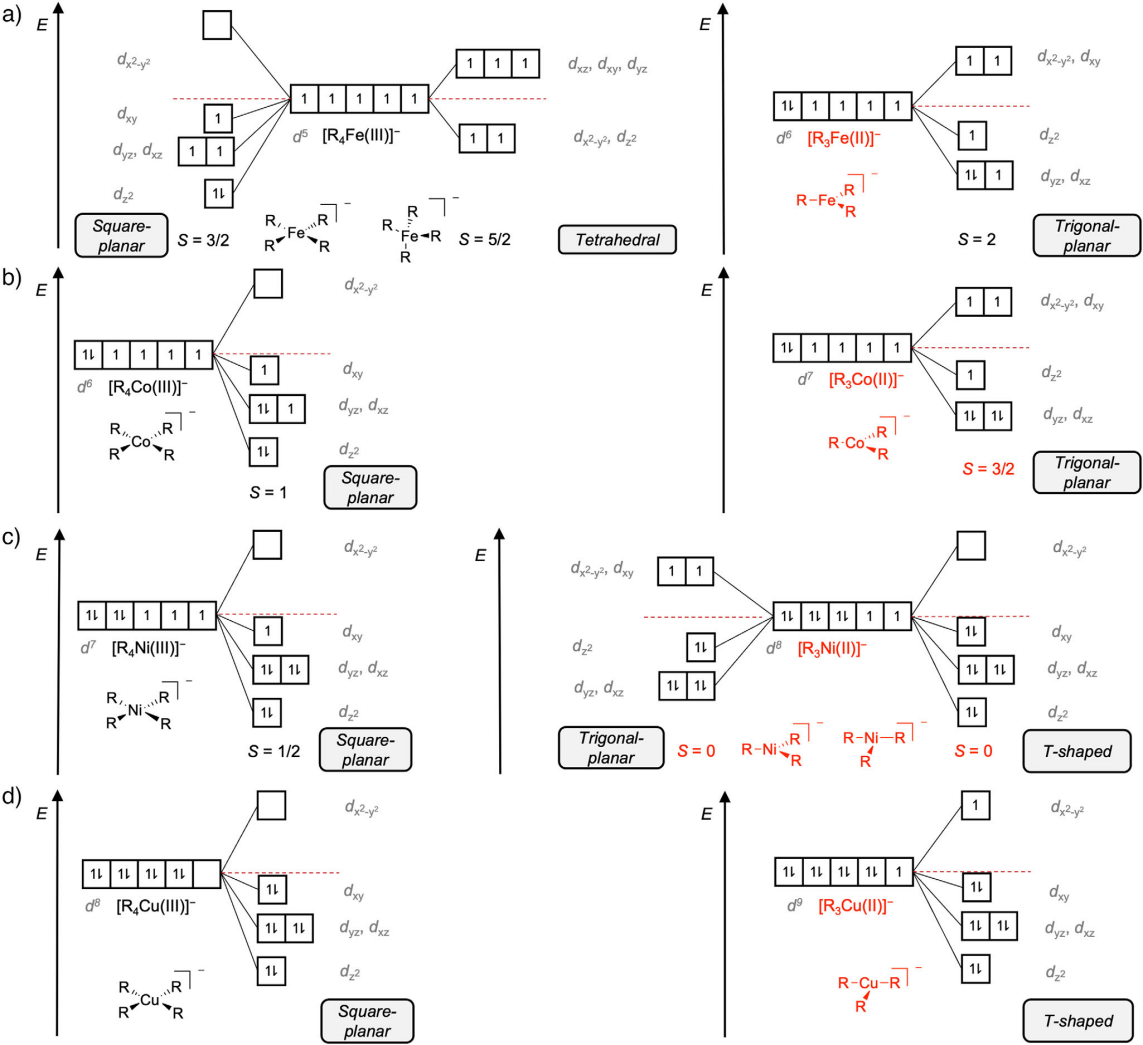
Qualitative MO diagrams for a) [R_4_Fe]^−^/[R_3_Fe]^−^, b) [R_4_Co]^−^/[R_3_Co]^−^, c) [R_4_Ni]^−^/[R_3_Ni]^−^, and d) [R_4_Cu]^−^/[R_3_Cu]^−^ in different spin states.

**[R_4_M]^−^**. The electronic ground states and structures of the [R*_n_* M]^−^ complexes were determined by quantum‐chemical calculations (see Supporting Information). These calculations predict a square planar ground‐state structure for all isolated [R_4_M]^−^ organometalates (see Supporting Information for xyz coordinates), which for iron is associated with an intermediate spin state (*S* = 3/2). With a d^5^ configuration, the energetically low‐lying orbitals of the Fe(III) complex are only incompletely occupied. Therefore, the energetic gain for the square‐planar geometry is only relatively small in this case as already a simple qualitative analysis of the energetic splitting of the d orbitals for this coordination environment indicates (Figure 1). This explains why the tetrahedral high‐spin configuration (*S* = 5/2) is computed to be only slightly higher in energy (+13.1 kJ mol^−1^). Previous analyses of tetraorganylferrates also suggest that the intermediate and high spin states are energetically quite close. The square‐planar geometry with the intermediate spin as ground state has been reported for [Me_4_Fe]^−^.^[^ ^38^ ^]^ In contrast, Mössbauer and EPR spectroscopy of [R_4_Fe]^−^, i.e., the very same complex as the one probed in the present work, pointed to a high‐spin ground state.^[^ ^39^, ^40^ ^]^ This result was also confirmed by theoretical calculations, which included the counter‐ion and a solvent molecule. Given the small energy difference between the two electronic states of [R_4_Fe]^−^, it may well be the case that their relative stability switches upon the transfer of the complex from the condensed phase into the gas phase.

For [R_4_Co]^−^, the presence of an additional d electron implies that the energy gain realized for a square‐planar coordination is higher than for its [R_4_Fe]^−^ congener. This qualitative assessment is in line with the prediction of this geometry with an intermediate spin (*S* = 1) being the ground state. It also agrees with previous results obtained for the related [Me_4_Co]^−^ complex.^[^ ^39^, ^40^ ^]^ The additional d electrons in [R_4_Ni]^−^ and [R_4_Cu]^−^ can also be filled into low‐lying orbitals within a square‐planar coordination geometry. Accordingly, we find the corresponding low‐spin electronic configurations to be most stable (*S* = 1/2 and *S* = 0, respectively), in accordance with similar results for analogous species reported in the literature.^[^ ^41^, ^42^ ^]^ Note that a more comprehensive analysis should take into account the possibility of so‐called inverted ligand fields, for which the metal‐based orbitals lie energetically below the ligand‐based ones.^[^ ^43^, ^44^ ^]^ As such a scenario does not change the spin state of the complexes and its consequences for the reactivity of the latter are not obvious,^[^ ^45^ ^]^ we do not further pursue this problem in the present context.

**[R_3_M]^−^**. For the [R_3_M]^−^ complexes, the quantum‐chemical calculations indicate two prevalent geometries: trigonal‐planar and T‐shaped (Figure 1). The ligand field of the latter resembles that of a square‐planar coordination, although the absence of the fourth ligand results in a smaller energy splitting. Like for their square‐planar counterparts, the occupation of the d orbitals of the T‐shaped complexes maximizes the number of paired electrons and, thus, gives rise to low‐spin configurations. Given the lower energy splitting in the T‐shaped ligand field, the resulting energetic gain is smaller than for the case of a square‐planar coordination. Accordingly, the [R_3_M]^−^ complexes are expected to have a decreased tendency to adopt low‐spin configurations. In line with this expectation, our calculations predict a high‐spin ground state (*S* = 2) with a trigonal‐planar geometry for [R_3_Fe]^−^. Previously, we found the same preference both theoretically and experimentally for other trisorganylferrates.^[^ ^25^, ^46^ ^]^ Similarly, the [R_3_Co]^−^ complex is computed to have a high‐spin ground state (*S* = 3/2) with a trigonal‐planar geometry. The preference of Co(II) species for a high‐spin configuration is well documented in the literature and has been subject to several studies due to the magnetic properties of these complexes.^[^ ^47^, ^48^, ^49^, ^50^ ^]^ For [R_3_Ni]^−^, we also find a high‐spin ground state (*S* = 1) with a trigonal‐planar geometry, although the low‐spin configuration (*S* = 0) with its T‐shaped structure is calculated to be only 4.3 kJ mol^−1^ higher in energy. Thus, both states are deemed as quasi‐degenerate and we expect a mixture of both to be present in our gas‐phase experiments. Similar behavior has previously been observed for other Ni(II) species. For instance, [((Me_3_Si)_2 _N)_3_Ni]^−^ features a trigonal‐planar geometry as predicted for the ground state of [R_3_Ni]^−^.^[^ ^51^ ^]^ Other cases of a facile conversion between high‐spin and low‐spin configurations are known and have been used for practical applications.^[^ ^52^, ^53^ ^]^ For [R_3_Cu]^−^, the d^9^ configuration necessarily leads to a low‐spin complex (*S* = 1/2), for which we predict a T‐shaped geometry. As the energetic demands for electron pairing does not differ for the two different geometries in this case, the placement of only 1 instead of 3 electrons in energetically high‐lying orbitals for the T‐shaped coordination geometry rationalizes the preference for the latter.

**[R_2_M]^−^**. All of the [R_2_M]^−^ complexes are found to adopt linear structures. The ferrate and cobaltate have intermediate spin states (with *S* = 3/2 and *S* = 1, respectively), whereas the nickelate and cuprate show low‐spin states (with *S* = 1/2 and *S* = 0). Thus, the electronic ground states of the individual [R_2_M]^−^ complexes correspond to those of their [R_4_M]^−^ counterparts.

### Overview of Dissociation Reactions

All accessible homoleptic mononuclear [R*_n_* M]^−^ metalates (M = Fe, Co, Ni, and Cu; *n* = 2–4) were mass selected and subjected to collision‐induced dissociation (CID) at different energies (Figures S7–S22). The observed fragmentation reactions correspond to the loss of R^•^ radicals, the reductive elimination of the R_2_ coupling product as well as the release of RH (Scheme 2). We show the normalized signal intensities of the different fragment ions as functions of the kinetic energy *E*_lab_ of the [R*_n_* M]^−^ precursor ions (so‐called breakdown curves, Figures S22–S35). The products of minor side reactions with residual traces of O_2_ or H_2_O present in the vacuum system are not included for better clarity. Complementary computational results are discussed alongside the experimental results to rationalize the observed fragmentation reactions.

**Scheme 2:**
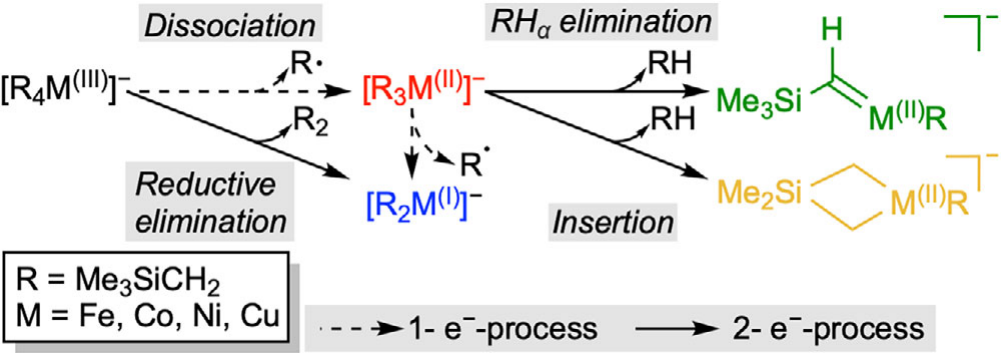
Dissociation reactions of [R*_n_* M]^−^ complexes.

### Dissociation of Organoferrates

**Figure 2:**
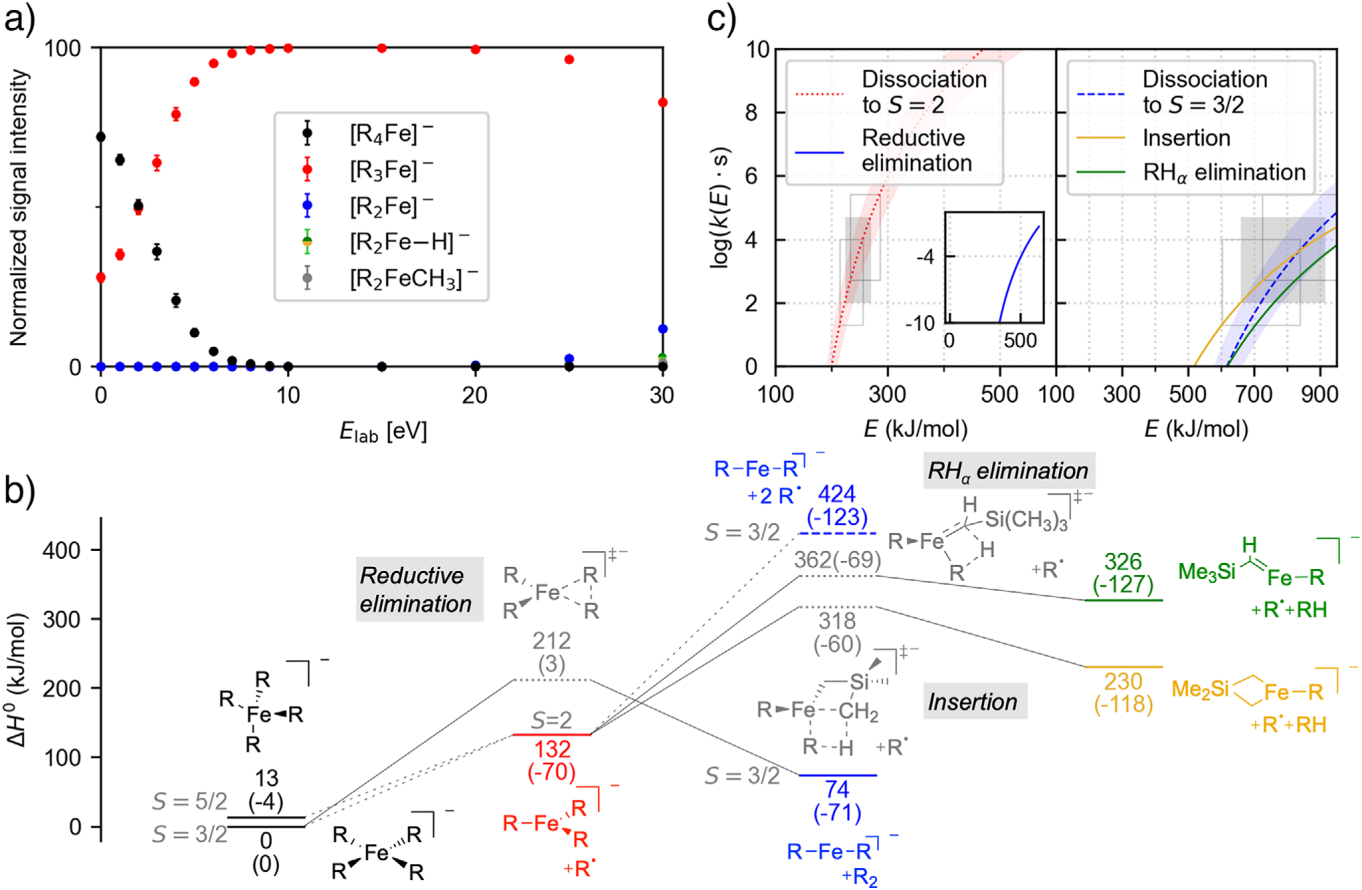
Experimental and computational results for the fragmentation of organoferrates. a) Breakdown curve for [R_4_Fe]^−^ (measurements for each data point in duplicate with given error bars corresponding to one standard deviation). b) DLPNO‐CCSD(T)//ωB97X‐D potential energy surfaces for the investigated spin states, with transition states indicated by gray, dotted bars. For all stationary points, relative enthalpies Δ*H*_0_ at 0 K are given on the respective bars. Relative entropies multiplied by −*T* for 298 K are given in parentheses. c) Microcanonical rate constants *k(E)* for the reaction pathways starting from the [R_4_Fe]^−^ intermediate‐spin complex, i.e., *S* = 3/2, (left) and the [R_3_Fe]^−^ high‐spin complex, i.e., *S* = 2, (right) based on the DLPNO‐CCSD(T)//ωB97X‐D results. For the energy‐independent rigidity factor *f*_rigid_, an uncertainty of log *f*_rigid_ = 2 ±1 is assumed. The filled gray boxes specify the estimated time scale of the experiment of *τ* = 10^−4^ s. The upper and lower empty gray boxes account for the uncertainty, which we estimate at *τ*_min_ = 10^−5^ s and *τ*_max_ = 5 × 10^−4^ s. A detailed explanation for this estimation is provided in the Supporting Information.

**[R_4_Fe]^−^**. Upon CID, the [R_4_Fe]^−^ ion mainly undergoes a homolytic bond cleavage to afford [R_3_Fe]^−^ (Figure 2a), as already reported previously.^[^ ^35^ ^]^ At higher energies, [R_2_Fe]^−^ emerges, which can result from the primary fragment ion [R_3_Fe]^−^ via the consecutive loss of another R^•^ radical or originate from the [R_4_Fe]^−^ precursor in a concerted reductive elimination. The fact that the signal intensity of [R_2_Fe]^−^ starts to rise when that of [R_3_Fe]^−^ begins to decrease indicates that the former is indeed produced by a consecutive radical loss from the latter. Furthermore, the observation of the fragment ions [R_2_Fe−H]^−^ and [R_2_FeMe]^−^ points to the loss of RH and the disintegration of a (trimethylsilyl)methyl ligand as minor processes.

According to the calculated DLPNO‐CCSD(T)//ωB97X‐D potential energy surface for the intermediate‐spin configuration, the barrier for the reductive elimination of R_2_ lies considerably higher (*H*_0_^‡^ = 211.8 kJ mol^−1^) than that for the loss of the R^•^ radical (132.4 kJ mol^−1^, Figure 2b). For the latter, we assume the barrier for the homolytic bond cleavage to be equivalent to the respective reaction enthalpy in the dissociation limit, because molecular ions typically do not feature a barrier for dissociation processes.^[^ ^54^ ^]^ The high‐spin configuration shows an even higher barrier for reductive elimination (see Table S4) and is therefore not considered any further.

Microcanonical rate constants, *k(E)*, were determined by means of Rice–Ramsperger–Kassel–Marcus (RRKM) theory based on the DLPNO‐CCSD(T)//ωB97X‐D potential energy surface for the homolytic bond cleavage and reductive elimination. For this purpose, we estimate the effective internal energy of the dissociating ions from the time window of the experiment (approximated as *τ* = 10^−4^ s).^[^ ^30^, ^55^, ^56^ ^]^ First, we assume that a fragmentation yield of ≥ 1% is needed to detect this process. Second, we consider the fragmentation complete if a total fragmentation yield of 99% for all reaction channels is reached. Thus, we derive rate constants of *k*(*E*) = 10^2^ s^−1^ and *k*(*E*) = 5 × 10^4^ s^−1^ as lower and upper limit, respectively. We explicitly account for the uncertainties of these values resulting from the assumed error bars of the experimental time window.^[^ ^57^ ^]^ The *k*(*E*) values for the homolytic bond cleavage rely on more assumptions than those of the two‐electron pathways due to the required estimation of an energy‐independent rigidity factor. Thus, a higher error can be assumed, which is indicated as the light shaded area in Figure 2c. The obtained results confirm the homolytic bond cleavage to furnish the high‐spin complex [R_3_Fe]^−^ (*S* = 2) as both the energetically and entropically preferred process. Accordingly, the only accessible gas‐phase fragmentation channel of [R_4_Fe]^−^ is the one‐electron pathway.

**[R_3_Fe]^−^**. In line with previous findings,^[^ ^35^ ^]^ the fragmentation of the mass‐selected [R_3_Fe]^−^ ion results in the loss of an R^•^ radical to form [R_2_Fe]^−^. This reactivity is fully consistent with the twofold homolytic bond cleavage inferred from the breakdown curve of [R_4_Fe]^−^ (see above). Additionally, we observe [R_2_Fe−H]^−^ and [R_2_FeCH_3_]^−^ as minor fragment ions. These ions are already known from the CID experiments of [R_4_Fe]^−^ and can be attributed to the fragmentation reactions outlined above. For the former, two different structures are conceivable: a carbene complex arising from the loss of an α‐hydrogen atom and a four‐membered cycle originating from the insertion of the iron center into a C─H bond in γ position (Scheme 2). To distinguish between these two possibilities, we performed a CID experiment on the [R_3_Fe]^−^ precursor ion with the (trimethylsilyl)methyl substituent deuterium‐labeled in γ position. We observed the elimination of both RH and RD with a slight preference for the latter (Figure S11). Accordingly, both the α elimination and the γ insertion must be operative simultaneously.

The quantum‐chemical computations predict the lowest enthalpic barrier *H*_0_^‡^ for the γ‐C─H insertion pathway (185.1 kJ mol^−1^), whereas the transition structures associated with the RH_α_ elimination (229.6 kJ mol^−1^) and, in particular, the radical dissociation (291.6 kJ mol^−1^) lie higher in energy (Figure 1). According to our calculated microcanonical rate constants, both the RH_α_ and RH_γ_ loss are accessible within the experimental time scale. In line with the deuterium‐labeling experiment, the γ‐C─H insertion pathway is preferred due to its lower barrier. Being entropically favored, the radical dissociation has a steeper *k*(*E*) curve such that it eventually outcompetes the two‐electron pathways at high energies.

**[R_2_Fe]^−^**. The [R_2_Fe]^−^ complex shows only a reaction with residual traces of O_2_ and/or H_2_O present in the vacuum system to furnish [Fe,C_3_,H_9_,Si,O]^−^. Although the exact structure of this fragment ion remains open, its generation obviously involves the disintegration of one of the (trimethylsilyl)methyl groups.

### Dissociation of Organocobaltates

**Figure 3:**
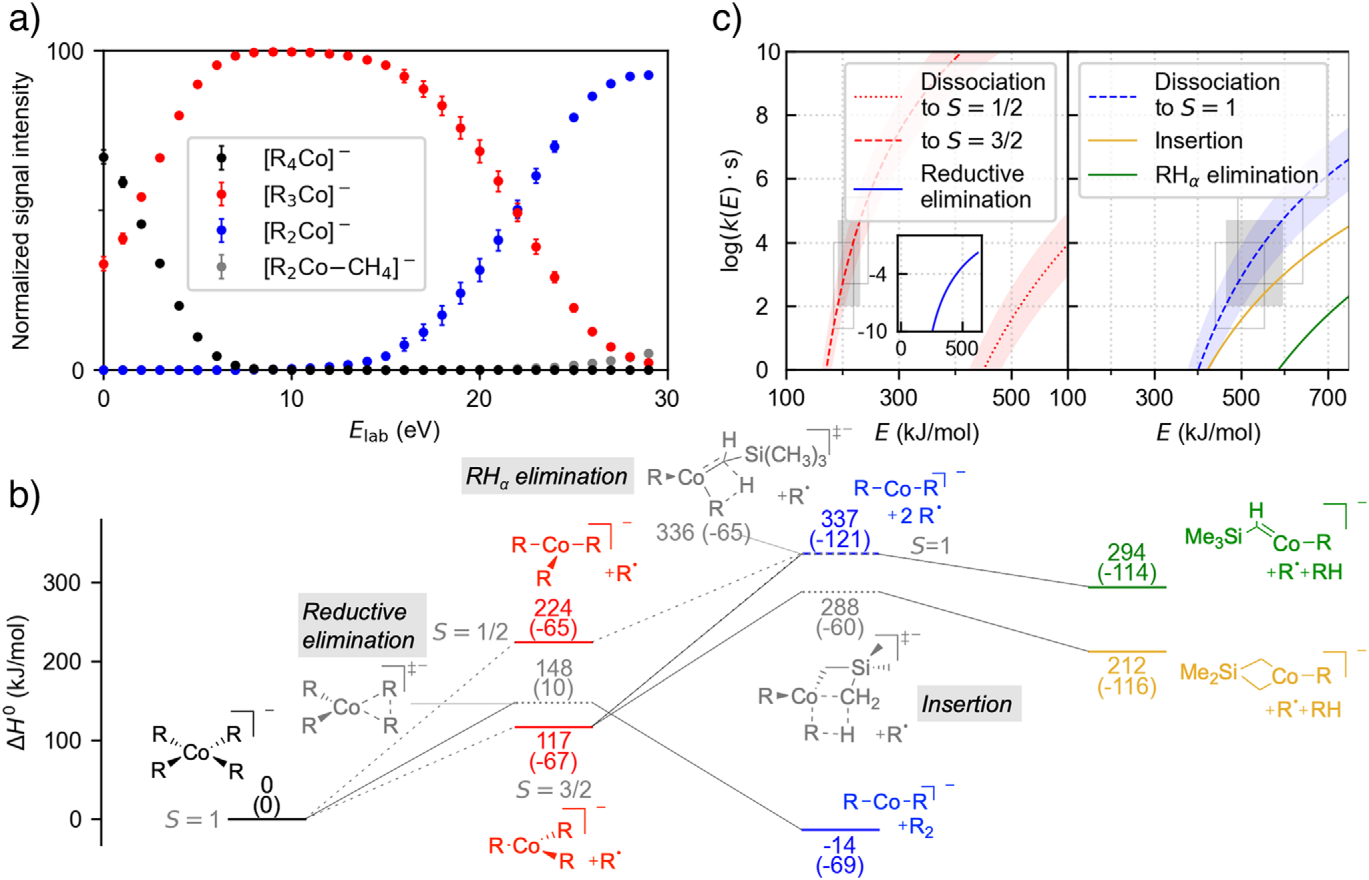
Experimental and computational results for the fragmentation of organocobaltates. a) Breakdown curve for [R_4_Co]^−^. b) DLPNO‐CCSD(T)//ωB97X‐D potential energy surfaces for the investigated spin states. c) Microcanonical rate constants *k(E)* for the reaction pathways starting from the [R_4_Co]^−^ intermediate‐spin (*S* = 1) complex (left) and the [R_3_Co]^−^ high‐spin (*S* = 3/2) complex (right) based on the DLPNO‐CCSD(T)//ωB97X‐D results. See Figure 2 for further details.

**[R_4_Co]^−^**. Similar to its organoferrate counterpart, [R_4_Co]^−^ mainly fragments into [R_3_Co]^−^ (Figure 3a). At higher collision energies, the fragment ions [R_2_Co]^−^ and [R_2_Co−CH_4_]^−^ also appear. In contrast to the fragmentation of [R_4_Fe]^−^, no RH loss occurs.

The calculated barrier for the reductive elimination of the intermediate‐spin complex [R_4_Co]^−^ (148.2 kJ mol^−1^) is lower than for the corresponding ferrate (Figure 3b). However, it is still higher than the enthalpy required for the dissociation of an R^•^ radical to afford [R_3_Co]^−^ in the *S* = 3/2 state (117.2 kJ mol^−1^). Our statistical rate‐theory calculations accordingly predict the predominance of radical dissociation over reductive elimination at the experimentally relevant energies (Figure 3c). Because of the entropic advantage of the dissociation pathway, even the formation of [R_3_Co]^−^ in the energetically unfavorable *S* = 1/2 state is kinetically preferred over reductive elimination.

**[R_3_Co]^−^**. Fragmentation of mass‐selected [R_3_Co]^−^ results predominantly in the release of an R^•^ radical, along with a small degree of (trimethylsilyl)methyl decomposition. These fragment ions are already known from the fragmentation of [R_4_Co]^−^ at high collision energies.

The lowest calculated barrier is that of the γ‐insertion pathway with 170.9 kJ mol^−1^. The competing radical dissociation exhibits an enthalpic barrier of 219.4 kJ mol^−1^, but is entropically favored. For this reason, it is calculated to prevail under the experimental conditions, in agreement with our observations (Figure 3c). According to predictions of the statistical rate‐theory calculations, the γ‐insertion is expected to occur to a minor extent, but was not observed in the experiments.

**[R_2_Co]^−^**. The [R_2_Co]^−^ complex undergoes multiple losses of CH_4_ to afford [R_2_Co−CH_4_]^−^ and [R_2_Co−2CH_4_]^−^. Furthermore, fragmentation of one the (trimethylsilyl)methyl groups results in [R_2_CoMe]^−^ (see Figure S14).

### Dissociation of Organonickelates

**Figure 4:**
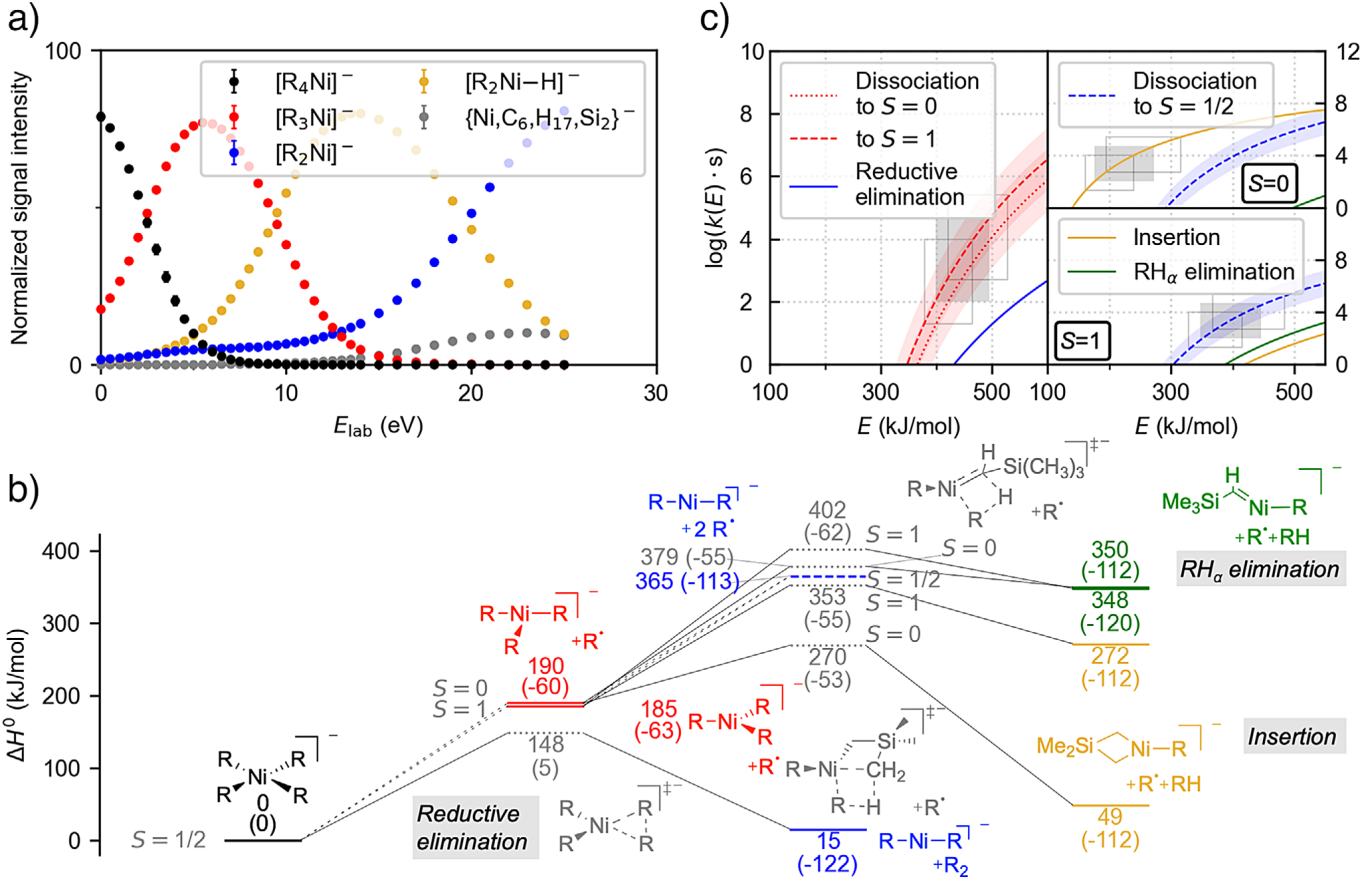
Experimental and computational results for the fragmentation of organonickelates. a) Breakdown curve for [R_4_Ni]^−^. b) DLPNO‐CCSD(T)//ωB97X‐D potential energy surfaces for the investigated spin states. c) Microcanonical rate constants *k(E)* for the reaction pathways starting from the [R_4_Ni]^−^ intermediate‐spin (*S* = 1/2) complex (left) and [R_3_Ni]^−^ (right) based on the DLPNO‐CCSD(T)//ωB97X‐D results. For [R_3_Ni]^−^, both the high‐ (*S* = 0) and low‐spin (*S* = 1) state are considered. See Figure 2 for further details.

**[R_4_Ni]^−^**. Like its iron‐and cobalt‐containing homologues, [R_4_Ni]^−^ loses an R^•^ radical to afford [R_3_Ni]^−^ at low collision energies (Figure 4a). Simultaneously, small amounts of [R_2_Ni]^−^ are formed. The signal intensity of the latter strongly increases at higher collision energies. This bimodal behavior seems to point to the operation of two different reaction pathways affording [R_2_Ni]^−^. The low‐energy reaction supposedly corresponds to the release of R_2_ in a primary process, whereas its high‐energy counterpart can be assigned to the consecutive loss of an R^•^ radical from the [R_3_Ni]^−^ intermediate. This analysis is complicated by the occurrence of another reaction channel, i.e., the formation of [R_2_Ni−H]^−^, which is the main fragment ion at medium collision energies. The competition between these different reaction pathways may possibly also result in a bimodal shape of the [R_2_Ni]^−^ breakdown curve. At high energies, [RNi{2C,6H,Si}]^−^ appears as yet another, though only minor fragment ion.

Our quantum‐chemical calculations predict the reductive elimination of [R_4_Ni]^−^ to be energetically least demanding (148.2 kJ mol^−1^). The loss of an R^•^ radical to afford high‐spin [R_3_Ni]^−^ (*S* = 1) is associated with a barrier of 185.4 kJ mol^−1^ (Figure 4b), with the low‐spin (*S* = 0) state being nearly degenerate. Because of the entropic preference of the radical‐liberation reaction, the latter is supposed to outcompete the reductive elimination (Figure 4c).

**[R_3_Ni]^−^**. Fragmentation of [R_3_Ni]^−^ results in the loss of RH and of an R^•^ radical, as was already deduced from the CID experiments on [R_4_Ni]^−^ (Figure 4a). Deuterium‐labeling experiments show that only the γ‐position is involved in formation of the [R_2_Ni−H]^−^ species. Thus, only the insertion pathway appears to occur for Ni. Additionally, decomposition of the (trimethylsilyl)methyl group is observed as a minor pathway.

As the *S* = 0 and the *S* = 1 spin state are calculated to be virtually degenerate, both of them are expected to be present in the experiments. However, these two electronic states are predicted to show different reactivity. The *S* = 0 state has a very low barrier of only 79.9 kJ mol^−1^ for the insertion pathway (see Figure 4b), whereas the radical‐dissociation threshold lies considerably higher (175.7 kJ mol^−1^) and is slightly below the barrier for RH_α_ elimination (189.0 kJ mol^−1^). For the *S* = 1 state, all three barriers are closer in energy with that of the insertion being the lowest (167.4 kJ mol^−1^), followed by those of the radical dissociation (180.1 kJ mol^−1^) and the RH_α_ elimination (217.1 kJ mol^−1^). The statistical rate‐theory calculations predict the γ‐insertion channel to be the preferred pathway for the *S* = 0 and the radical dissociation for the *S* = 1 state (Figure 3c). Although the latter reaction benefits from a strong entropic advantage in both cases, the enthalpic preference for the γ‐insertion channel is so large in the case of the low‐spin state that this pathway is predicted to prevail for this complex in the accessible energy window. Thus, the observation of the two competing reaction channels gives strong evidence for the population of the two nearly degenerate spin states of the [R_3_Ni]^−^ complex in the experiment.

**[R_2_Ni]^−^**. CID of [R_2_Ni]^−^ gives rise to the fragment ions [R_2_Ni−CH_4_]^−^, [R_2_Ni−2CH_4_]^−^, and [R_2_NiMe]^−^ (Figure S17). This behavior resembles that of the homologous [R_2_Co]^−^ species (see above).

### Dissociation of Organocuprates

**Figure 5:**
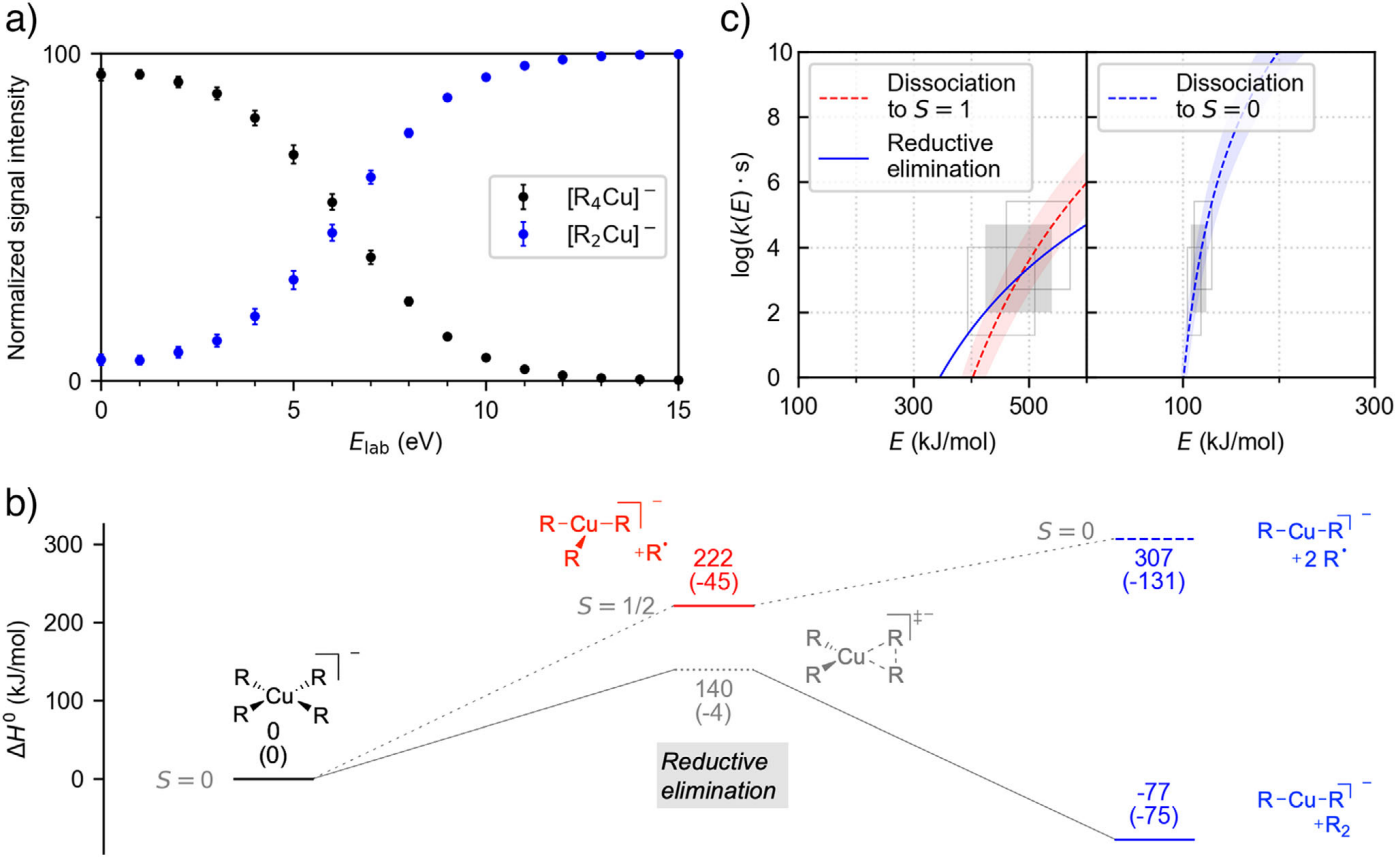
Experimental and computational results for the fragmentation of organocuprates. a): Breakdown curve for [R_4_Cu]^−^. b) DLPNO‐CCSD(T)//ωB97X‐D potential energy surfaces for the investigated spin states. c) Microcanonical rate constants *k(E)* for the reaction pathways starting from [R_4_Cu]^−^ (*S* = 0, left) and [R_3_Cu]^−^ (*S* = 1/2, right) based on the DLPNO‐CCSD(T)//ωB97X‐D results. See Figure 2 for further details.

**[R_4_Cu]^−^**. CID of [R_4_Cu]^−^ exclusively furnishes the fragment ion [R_2_Cu]^−^ (Figure 5a). This observation implies the operation of a two‐electron reductive elimination or of two fast consecutive radical R^•^ dissociations, with the lifetime of the hypothetical [R_3_Cu]^−^ intermediate being in the ms range at most.

We calculate a barrier of 139.5 kJ mol^−1^ for the concerted reductive elimination of [R_4_Cu]^−^ (Figure 5b). This value is the lowest for all organometalates [R_4_M]^−^ considered in the present work. It lies significantly below the enthalpy associated with the loss of an R^•^ radical (221.8 kJ mol^−1^). In contrast, the dissociation of an R^•^ radical from the hypothetical [R_3_Cu]^−^ intermediate requires only 85.5 kJ mol^−1^. As already demonstrated for the other complexes, the relative efficiency of the competing reaction channels is controlled both by enthalpic and entropic factors, the latter of which consistently favor the radical dissociations. In the case of [R_4_Cu]^−^, our statistical rate‐theory calculations predict very similar rate constants for the concerted reductive elimination and the radical loss within the experimentally relevant energy window (Figure 5c). Reductive elimination dominates for the lower half of the experimentally accessed energy window, whereas radical dissociation is predominant at higher energies. The resulting [R_3_Cu]^−^ ion very efficiently releases another R^•^ radical, which fully accounts for the fact that we could not observe it in our experiments.

**[R_2_Cu]^−^**. Upon fragmentation, the [R_2_Cu]^−^ complex exclusively yields [RCuMe]^−^ (Figure S22). Analogous reactions occur for [R_2_Co]^−^ and [R_2_Ni]^−^ (see above).

### Trends in the Reactivity of [R*_n_* M]^−^ Complexes

**Figure 6:**
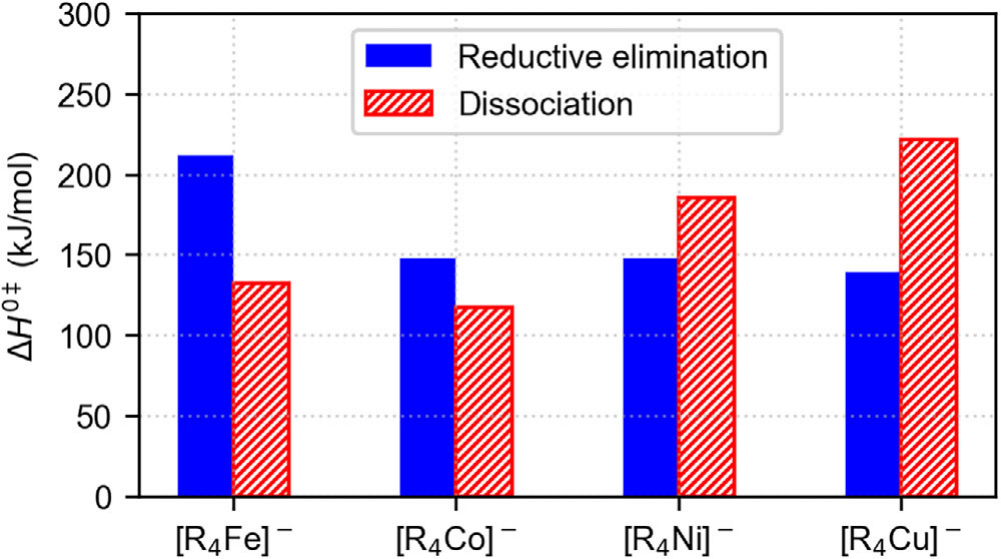
Barriers associated with the concerted reductive elimination of R_2_ (blue) and the loss of an R^•^ radical (red) from [R_4_M]^−^. The latter are supposed to equal the bond‐dissociation energies.

**[R_4_M]^−^**. The probed [R_4_M]^−^ complexes show a clear trend in their unimolecular reactivity: The tendency toward a concerted reductive elimination, i.e., a two‐electron process, increases in the series M = Fe, Co, Ni, and Cu. While [R_4_Fe]^−^ and [R_4_Co]^−^ undergo only homolytic bond cleavages and, thus, one‐electron processes, the concerted reductive elimination apparently starts to compete for [R_4_Ni]^−^ and occurs for [R_4_Cu]^−^ to a large degree. As outlined above, the energetic preference for the concerted reductive elimination (ΔΔ*H* ^‡^ values) is even more pronounced (Figure 6) but is counteracted by the entropically unfavorable highly‐ordered nature of its transition structure.

**Figure 7:**
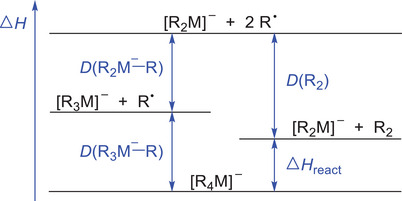
Thermochemical cycle relevant to the unimolecular reactivity of [R_4_M]^−^.

For an analysis of the origin of this trend in the barriers, we adopt an approach following Marcus theory^[^ ^58^ ^]^ and distinguish between the thermodynamic driving force Δ*H*_react_ and the intrinsic barrier of the reactions in question. A simple thermochemical cycle shows that the reaction energy of the concerted reductive elimination of R_2_ from [R_4_M]^−^ equals the sum of the first and second dissociation energy of R^•^ radicals minus the bond‐dissociation energy of the R_2_ coupling product (Figure 7). The latter remains constant for the whole series of reactions analyzed here and, thus, does not need to be considered for rationalizing the relative trend.

**Figure 8:**
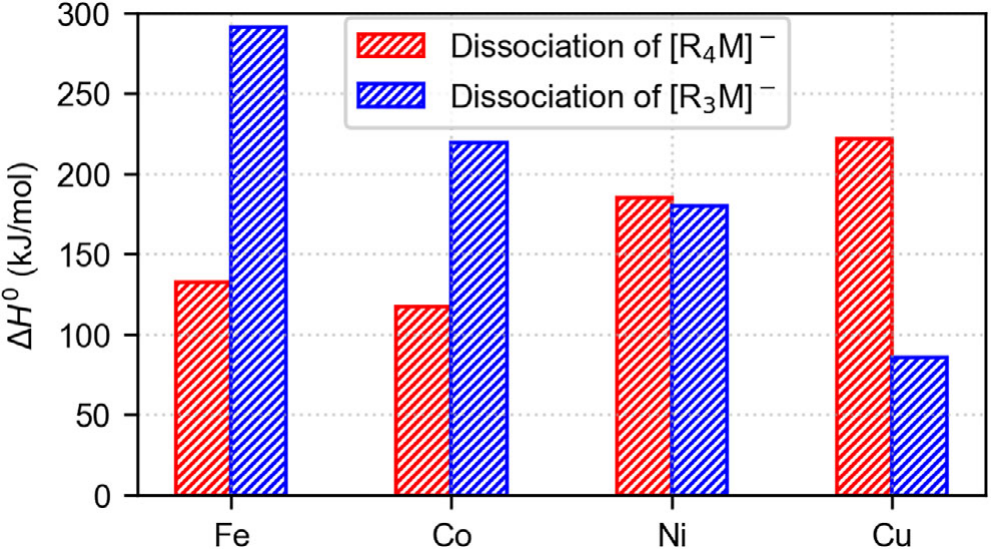
First (red) and second (blue) bond‐dissociation energies of [R_4_M]^−^.

As a first (simplistic) approximation, we moreover assume that the intrinsic barriers associated with the concerted reductive eliminations do not strongly change for the different metals. In this case, only the sum of the first and second *D*(R*_n_* M^−^−R) dissociation energy matters. Thus, a higher first *D*(R*_n_* M^−^−R) dissociation energy not only makes the homolytic bond cleavage more difficult, but also raises the barrier for the concerted reductive elimination. For the competition between the two pathways, the ratio between first and second *D*(R*_n_* M^−^−R) dissociation energy turns out to be decisive. If the first dissociation requires less energy than the second, the one‐electron pathway can proceed relatively easily, whereas the increased barrier of the concerted reductive elimination prevents the latter reaction. Such a scenario is realized for [R_4_Fe]^−^ and [R_4_Co]^−^ (Figure 8). In contrast, if the first dissociation requires more energy than the second, the initial homolytic bond cleavage is hindered. The concerted reductive elimination can still take place because the barrier of this pathway is lowered by binding interactions between the two R^•^ groups in the transition structure. Such a situation is found for [R_4_Cu]^−^. For [R_4_Ni]^−^, the first and second R^•^ dissociation energies are calculated to be almost equal. In line with this prediction, both the one‐and two‐electron pathway seem to occur, although the former is strongly favored on entropic grounds (see above).

The different ratios in the first and second R^•^ dissociation energies as the controlling factors in the reactivity of the [R_4_M]^−^ complexes reflect the deviating relative stabilities of the different oxidation states of the 3d metals. For Fe and Co, the electronegativity does not appear to be high enough to stabilize the high electron density at the M(I) center of the [R_2_M]^−^ fragment ions sufficiently. Although stable complexes of these metals in low oxidation states are well known,^[^ ^36^, ^59^, ^60^ ^]^ they require the presence of ligands capable of abstracting electron density by backbonding, unlike the Me_3_SiCH_2_ group. In contrast, the higher electronegativity of Ni and, in particular, Cu renders the M(I) oxidation state much more stable as the well‐established chemistry of organocuprates(I) clearly demonstrates. These Cu(I) species with their linear coordination geometries also benefit from the special stability of this configuration, in which the energetic cost of the sd hybridization state is shared between the two ligands.^[^ ^61^ ^]^ The scarcity of organocopper complexes in oxidation state II mirrors the low stability of these species, whose d^9^ configuration implies that one of the d electrons populates an energetically high‐lying orbital.

As our analysis has shown, the relevant thermochemistry is also influenced by the bond‐dissociation energy of the R_2_ coupling product: The higher this bond‐dissociation energy, the more stable the products of the concerted reductive elimination.^[^ ^62^ ^]^ The calculated bond‐dissociation energy of R_2_, R = Me_3_SiCH_2_, *D*(R─R) = 350.2 kJ/mol, is somewhat lower than that of most other hydrocarbons with sp^3^‐hybridized carbon atoms. Thus, in this case, the thermochemical driving force for the concerted reductive elimination is diminished accordingly. This decreased preference for concerted reductive eliminations is reflected in the predominance of radical pathways observed in the present work. The opposite behavior is expected for reactions resulting in R_2_ coupling products with increased bond‐dissociation energies, such as bisaryls, in which the bond to be cleaved involves two sp^2^‐hybridized carbon atoms.

Although the analysis of the relevant thermochemistry seems quite helpful and instructive, the neglect of differences of the intrinsic barriers remains a simplification. Our calculations permit us to estimate the error introduced by this assumption. For M = Fe, Co, and Ni, we predict intrinsic barriers associated with the reductive elimination of R_2_ in the range of 147 ± 15 kJ mol^−1^. Thus, the variation indeed is relatively small and, thus, can be neglected to a first approximation. This finding seems in line with the chemical similarity of iron, cobalt, and nickel, which is commonly observed and originally led to the classification of these elements into a single group (group VIII). In contrast, the intrinsic barrier derived for the concerted reductive elimination for M = Cu amounts to 217 kJ mol^−1^. This increased barrier implies that the concerted reductive elimination proceeds less efficiently than expected on the basis of the involved thermochemistry. Hence, it also rationalizes the inferred occurrence of radical dissociations along with concerted reductive eliminations for the case of [R_4_Cu]^−^.

**[R_3_M]^−^**. None of the probed [R_3_M]^−^ complexes is found to undergo a concerted reductive elimination. Instead, these species predominantly react via the dissociation of R^•^ radicals. In addition, the [R_3_M]^−^ complexes undergo varying degrees of disintegration of the Me_3_SiCH_2_ group. Such a reactivity is most pronounced for the *S* = 0 spin state of [R_3_Ni]^−^, for which the insertion of the metal center into a γ‐C─H bond outcompetes the radical dissociation. This selectivity deviates from that of the corresponding *S* = 1 spin state, which preferentially reacts via radical dissociation. Thus, this case clearly demonstrates that complexes in lower spin states tend to prefer two‐electron pathways whereas the opposite holds true for their high‐spin congeners.

**[R_2_M]^−^**. The [R_2_M]^−^ complexes undergo neither concerted reductive eliminations nor radical dissociations, but various reactions resulting in the disintegration of the Me_3_SiCH_2_ group. This reactivity shows the high tendency of these species to increase their coordination numbers and fill open coordination sites.

### Comparison With Previous Findings

The present results suggest that the relative size of the sequential M─organyl bond‐dissociation energies is essential for controlling the competition between one‐ and two‐electron processes of organometallic complexes. Unfortunately, a more general test of this rationale is not possible due to the lack of quantitative data on M─organyl bond strengths. Nonetheless, the tendency of the late 3d metals for one‐ as well as two‐electron reactions is well‐documented. For iron, numerous previous studies point to the importance of radical processes.^[^ ^12^, ^63^, ^64^ ^]^ Homolytic bond cleavages have been invoked as key steps in numerous iron‐catalyzed transformations in the condensed phase, such as cross‐coupling and C─H activation reactions.^[^ ^65^, ^66^, ^67^, ^68^, ^69^, ^70^, ^71^ ^]^ Even more detailed information can be derived from previous gas‐phase experiments, which, like our present work, probe organoferrates in different oxidation states. In line with the present results, these experiments find a high propensity of tetra‐alkylferrates(III) to radical dissociations, i.e., one‐electron reactions.^[^ ^35^ ^]^ In comparison, trialkylferrates(II) can also undergo two‐electron processes, such as β‐hydrogen eliminations. This finding is fully consistent with the observation of RH_α_ elimination and γ‐insertion reactions as minor two‐electron processes for [R_3_Fe]^−^ in the present case. For aryl‐rich organoferrates, the reactivity completely shifts toward two‐electron processes, such as concerted reductive eliminations for Fe(III) and arene losses for Fe(II) species.^[^ ^33^, ^35^ ^]^ This change in reactivity reflects the higher stability of the thus‐formed coupling products and the avoidance of unstable aryl radicals in accordance with the thermochemical analysis presented above.

Like iron, cobalt is well‐known for its ability to form radicals via the homolytic cleavage of Co─C bonds, even under mild conditions.^[^ ^72^ ^]^ Thus, most cobalt‐catalyzed reactions are assumed to proceed via one‐electron pathways,^[^ ^73^ ^]^ which agrees with the predominance of radical dissociations observed in the present study. The preference of cobalt and iron for one‐electron processes also explains why catalytic applications of these metals are usually not severely impeded by β‐H elimination, a two‐electron reaction notorious for plaguing palladium‐catalyzed transformations involving alkyl systems.^[^ ^74^, ^75^, ^76^, ^77^ ^]^ To which extent the release of coupling products from high‐valent cobalt centers proceeds in a stepwise (one‐electron pathway) or concerted fashion (two‐electron pathway), remains largely unknown although the occurrence of concerted reductive eliminations has been frequently proposed.^[^ ^78^, ^79^, ^80^, ^81^ ^]^ The present results suggest that at least sp^3^‐hybridized organyl groups bound to a Co(III) center preferentially react stepwise via radical intermediates.

Our present findings show that nickel exhibits a borderline reactivity including both one‐and two‐electron reactions, which renders it particularly interesting for catalysis. This special reactivity of nickel indeed is exploited in current nickel‐catalyzed cross‐coupling reactions. Although Ni(0), like Pd(0), easily undergoes oxidative addition followed by transmetalation, the resulting Ni(II) complexes have only a rather low tendency toward reductive elimination. The combination of Ni catalysis with photoredox catalysis overcomes this problem because the excited photocatalyst oxidizes the Ni(II) complex to afford the corresponding Ni(III) species in a single‐electron transfer, i.e., a typical one‐electron process.^[^ ^17^, ^82^, ^83^, ^84^ ^]^ The thus formed Ni(III) complex then readily undergoes a concerted reductive elimination of the coupling product, i.e., a two‐electron process. Another mechanistic pathway involves the generation of electronically excited Ni(II) species.^[^ ^82^, ^83^, ^84^ ^]^ The feasibility of this pathway reflects the availability of different electronic states for nickel, which the present calculations also highlighted. Before the advent of nickel photoredox catalysis, examples of nickel involved in one‐ and/or two‐electron reactions had also been known.^[^ ^17^, ^85^, ^86^, ^87^ ^]^

The reactivity of organocopper species has long been considered to be limited to two‐electron processes,^[^ ^88^, ^89^, ^90^, ^91^, ^92^, ^93^ ^]^ with Cu(I) and Cu(III) as the only relevant oxidation states although the stability of nonorganometallic Cu(II) complexes in aqueous solution is common knowledge.^[^ ^94^ ^]^ Cu(III) species were found to undergo reductive eliminations very easily, which is why their observation and characterization proved challenging for a long time.^[^ ^95^, ^96^, ^97^ ^]^ The ability and readiness of Cu(III) species to react via reductive eliminations is fully consistent with the present results. However, our calculations suggest that only a part of the probed [R_4_Cu]^−^ complexes undergo a concerted reductive elimination, whereas the other fraction reacts in a sequence of radical dissociations. The lifetime of the [R_3_Cu]^−^ intermediate appears to be so short that not even the present gas‐phase experiments with their ms‐time resolution succeeded in their detection. Interestingly, analogous experiments intercepted Cu(II) fragment ions resulting from the fragmentation of [(CF_3_)_4_Cu]^−^,^[^ ^98^, ^99^ ^]^ [(CF_3_)_3_Cu(alkyl)]^−^,^[^ ^30^ ^]^ and [Me_3_Cu(allyl)]^−^.^[^ ^31^ ^]^ Thus, these gas‐phase experiments established the viability of stepwise radical dissociations from high‐valent organocopper species. Very recently, the occurrence of such processes in the condensed phase could also be demonstrated.^[^ ^100^ ^]^ It seems quite likely that one‐electron reactions are much more important in organocopper chemistry than previously recognized.

## Conclusion

Using a combination of tandem‐mass spectrometry and quantum‐chemical calculations, we have characterized the unimolecular reactivity of the late 3d‐metal complexes [R*_n_* M]^−^ (M = Fe, Co, Ni, and Cu; *n* = 2–4). These model systems showed the competition between one‐ and two‐electron processes typical of 3d‐metal chemistry and permitted the identification of a clear trend. While barrierless dissociations of R^•^ radicals, i.e., one‐electron reactions, predominate for [R_4_Fe]^−^ and [R_4_Co]^−^, concerted reductive eliminations of the R_2_ coupling product, i.e., two‐electron reactions, start to compete for [R_4_Ni]^−^and further increase in importance for [R_4_Cu]^–^. This trend is chiefly controlled by the relative order of the *D*(R_3_M^−^−R) and *D*(R_2_M^−^−R) bond‐dissociation energies. If the latter exceed the former, radical losses prevail, whereas concerted reductive eliminations are energetically preferred for the reverse order. The relative bond‐dissociation energies, in turn, largely reflect the stability of the metal centers in their different oxidation states. We expect similar behavior for other series of related 3d‐metal complexes. Additional effects are brought about by entropic and kinetic factors. Entropically, the radical dissociations are more favorable than the concerted reductive eliminations with their highly ordered transition structures. Kinetic factors result in a distinction between [R_4_Fe]^–^, [R_4_Co]^–^, and [R_4_Ni]^–^ on the one hand and [R_4_Cu]^–^ on the other. The former complexes all exhibit similar intrinsic barriers associated with the concerted reductive elimination, whereas that of the latter is significantly higher. Thus, the cuprate has a lower tendency toward the two‐electron pathway than expected on thermodynamic grounds. This peculiarity explains why the one‐electron reaction can compete in this instance as well as in other cases of high‐valent organocopper species recently reported in the literature.

Besides radical dissociations and reductive eliminations, we identified additional reactions, such as RH_γ_ insertion and RH_α_ elimination as possible fragmentation pathways. For the detailed elucidation of these processes, deuterium‐labeling experiments along with further computational investigations were essential. Of all systems probed, the nickelate complex exhibited the most diverse reactivity because the [R_3_Ni]^−^ species formed after the initial R^•^ radical dissociation has two degenerate spin states (*S* = 0 and *S* = 1), which react toward different products.

The obtained good agreement between experiment and theory required the application of highly accurate electronic‐structure methods and an involved kinetic analysis. The latter, in particular, proved necessary in the case of the radical dissociation reactions, whose strong entropic effects render an analysis simply based on enthalpy barriers absolutely insufficient. Although efficient radical recombination pathways and solvent effects certainly change the net reactivity of related complexes in solution, the present gas‐phase results provide direct insight into the intrinsic reactivity of late 3d‐metal complexes. As such, they may serve as an unbiased reference for analyzing and predicting trends in synthetic and catalytic applications of these systems. In this respect, the different propensities of the individual 3d metals for one‐ and two‐electron reactions revealed here are of prime importance and offer themselves for practical exploitations.

## Supporting Information

The authors have cited additional references within the Supporting Information.^[^ ^54^, ^101^, ^102^, ^103^, ^104^, ^105^, ^106^, ^107^, ^108^, ^109^, ^110^, ^111^, ^112^, ^113^, ^114^, ^115^, ^116^, ^117^, ^118^, ^119^ ^]^

## Conflict of Interests

The authors declare no conflict of interest.

## Supporting information



Supporting Information.

## Data Availability

The data that support the findings of this study are available in the supplementary material of this article.
