# Supplementary material for: Competition between One‐ and Two‐Electron Unimolecular Reactions of Late 3d‐Metal Complexes [(Me3SiCH2) n M]–(M = Fe, Co, Ni, and Cu; n = 2–4)
Source: Angew Chem Int Ed Engl. 2025 Apr 4;64(21):e202500524. doi: 10.1002/anie.202500524 (PMC12087865; doi:10.1002/anie.202500524)
Supplement: Supplementary file 1 — Supporting Information. [file ANIE-64-e202500524-s001.pdf]

Table of Contents

1 Experimental details *2*

1.1 Sample preparation 2

1.2 Mass-spectrometric measurements 2

2 Additional mass spectra *3*

2.1 ESI-mass spectra 3

2.2 List of recorded ions 5

2.3 CID spectra 6

2.4 Additional breakdown curves 12

3 Computational methods *17*

4 Results of electronic structure calculations *18*

4.1 Optimized structures 18

4.2 Energetics 18

1 Experimental details

1.1 Sample preparation

All samples were prepared at the concentrations given in **Table S1** at a scale that resulted in 5 mL of sample solution. All metal salts were dried under vacuum under the conditions listed in **Table S1** before being dissolved or suspended in water-free THF. Afterwards, the transmetalation reagent was added at −78 °C.

Table S1: Metal salts and preparation conditions.

Metal Salt	Drying Procedure	Concentration (metal ion)	Transmetalation reagent
$Fe(acac)_3$	80 °C, vac., 10 min	5 mmol/L	4 eq. Me_3SiCH_2Li
$Fe(acac)_3$	80 °C, vac., 10 min	10 mmol/L	4 eq. $(CD_3)_3SiCH_2Li$
$CoCl_2$	250 °C, vac., 1 h	10 mmol/L	4 eq. Me_3SiCH_2Li
$Ni(acac)_2$	100 °C, vac., 30 min	5 mmol/L	4 eq. Me_3SiCH_2Li
$Ni(acac)_2$	100 °C, vac., 30 min	5 mmol/L	4 eq. $(CD_3)_3SiCH_2Li$
Cu(OAc)	100 °C, vac., 15 min	10 mmol/L	4 eq. Me_3SiCH_2Li

1.2 Mass-spectrometric measurements

Sample solutions (5 or 10 mM) were prepared by dissolution of the corresponding metal salt in THF followed by the addition of 4 eq. of RLi at 195 K under inert conditions. Samples were introduced directly from the cooled flask into the ESI source (3.5 kV) of a micrOTOF-Q II mass spectrometer (Bruker Daltonik, Bremen, Germany) via pressurized sample injection (27 mbar backing pressure, 0.5 mm inner-diameter tubing, 50 cm tube length).[1] Nitrogen was used as the nebulizer (0.7 bar) and the drying gas (6 L/min, 333 K). The detailed instrument settings are listed in **Table S2**. CID spectra for breakdown curves were recorded for kinetic energies E_{lab} of up to 30 eV, depending on the observed reactivity and achievable fragment ion intensities. Measurements were performed in duplicate for each data point and the normalized signal intensities of the fragment ions were averaged. Ions resulting from reactions with residual traces of O_2 or H_2O were not considered. The shown error bars correspond to one standard deviation.

Table S2: Settings used on the Bruker micrOTOF-Q II mass spectrometer.

Ion Source		Ion Transfer	
End Plate Offset	500 V	Funnel 1 RF	200.0 Vpp
Capillary	3000 V	Funnel 2 RF	400.0 Vpp
Nebulizer	10.0 psi	isCID Energy	0.0 eV
Dry Gas	6.0 L/min	Hexapole RF	200.0 Vpp
Dry Temperature	60 °C		
**Quadrupole**		**Collision Cell**	
Collision Energy	0.0 eV	Ion Energy	5.0 eV
Collision RF	200.0 Vpp	Low Mass	200.0 m/z
Transfer Time	50.0 µs		
Pre Pulse Storage	10.0 µs		
		Collision Gas (N_2) Flow	15.0 %

2 Additional mass spectra

2.1 ESI-mass spectra

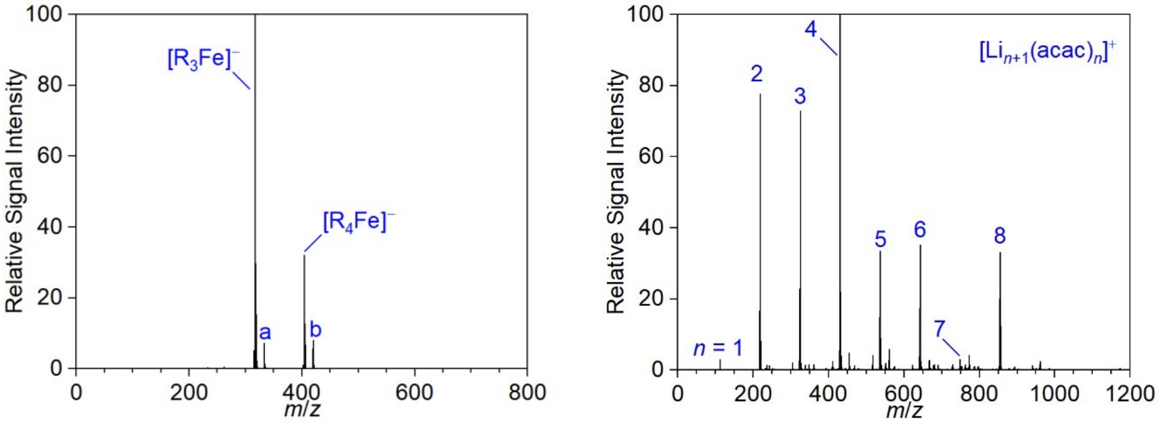

Figure S1. ESI-mass spectra of the products resulting from the reaction of $Fe(acac)_3$ and 4 eq. of Me_3SiCH_2Li in THF measured in the negative (left, a = $\{Fe,R_3,O\}^-$, b = $\{Fe,R_4,O\}^-$) and positive ion mode (right), R = Me_3SiCH_2.

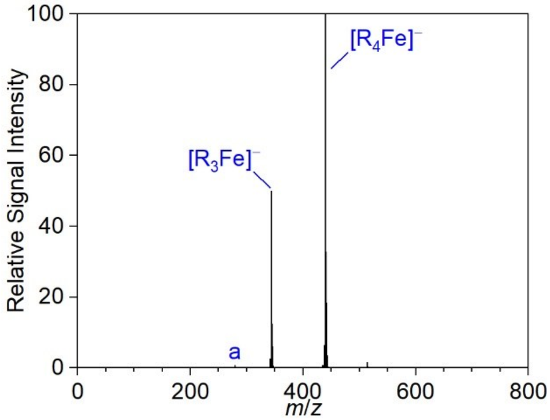

Figure S2. Negative-ion mode ESI-mass spectrum of the products resulting from the reaction of $Fe(acac)_3$ with 4 eq. of $(CD_3)_3SiCH_2Li$ in THF (a = $\{Fe,R_2,O_2\}^-$), R = $(CD_3)_3SiCH_2$).

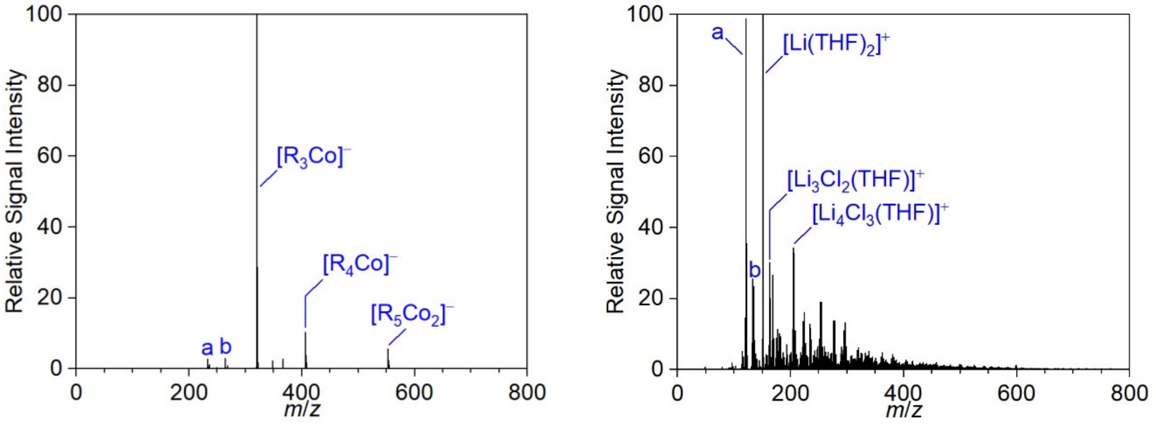

Figure S3. ESI-mass spectra of the products resulting from the reaction of $CoCl_2$ with 4 eq. in THF measured in the negative (left, a = $[R_2Co]^-$, b = $\{Co,R_2,O_2\}^-$) and positive ion mode (right, a = $[Li_2Cl(THF)]^+$, b = $[Li_4Cl_3]^+$), R = Me_3SiCH_2.

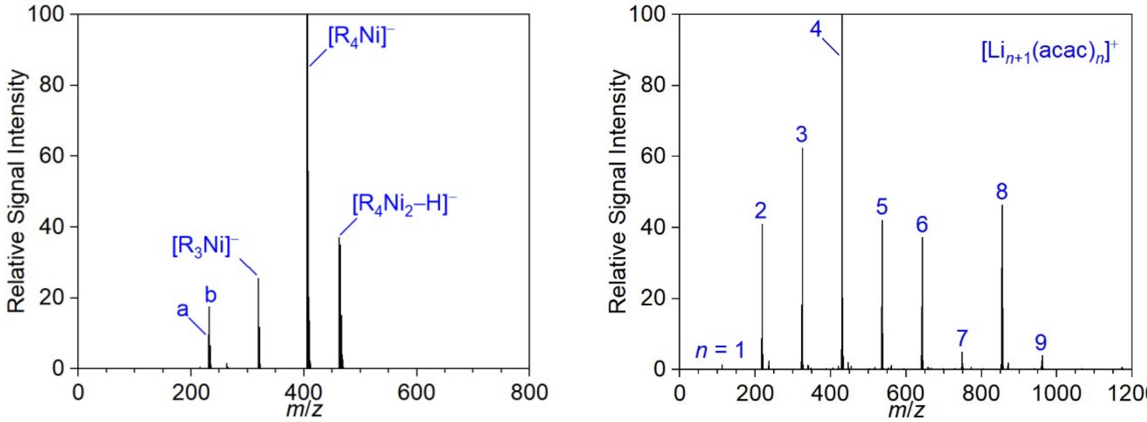

Figure S4. ESI-mass spectra of the products resulting from the reaction of $Ni(acac)_2$ with 4 eq. of Me_3SiCH_2Li in THF measured in the negative (left, a = $[R_2Ni{-}H]^-$, b = $[R_2Ni]^-$) and positive ion mode (right), R = Me_3SiCH_2.

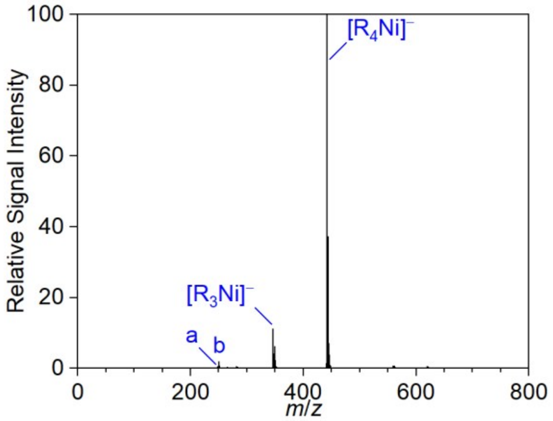

Figure S5. Negative-ion mode ESI-mass spectrum of the products resulting from the reaction of $Ni(acac)_2$ with 4 eq. of $(CD_3)_3SiCH_2Li$ in THF (a = $[R_2Ni{-}D]^-$, b = $[R_2Ni]^-$), R = $(CD_3)_3SiCH_2$.

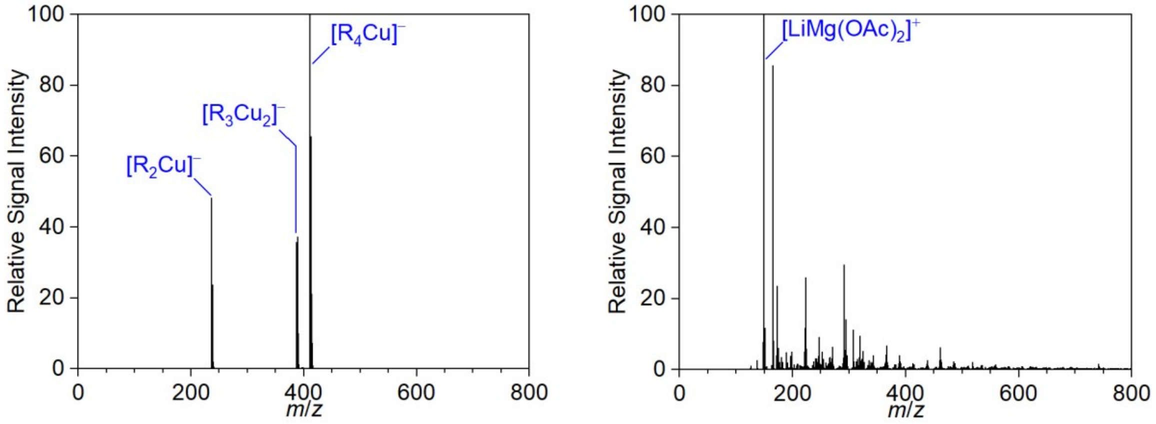

Figure S6. ESI-mass spectra of the products resulting from the reaction of CuOAc with 4 eq. of Me_3SiCH_2Li in THF measured in the negative (left) and positive ion mode (right), R = Me_3SiCH_2.

2.2 List of recorded ions

Table S3. List of observed anions in ESI-mass spectra and their fragment/product ions formed upon CID, R = Me_3SiCH_2.

Ion	Fragment/Product ions
$[R_2Fe]^-$	$\{Fe,C_3,H_9,Si,O\}^-$
$[R_3Fe]^-$	$\{(Fe,R,H,O){-}CH_3\}^-$, $[R_2Fe{-}H]^-$, $[R_2Fe]^-$, $[R_2FeCH_3]^-$, $\{Fe,R_2,O_2\}^-$, *m/z* = 146.92 (unassigned)
$\{Fe,R_3,O\}^-$	
$[R_4Fe]^-$	$\{(Fe,R,O,H){-}CH_3\}^-$, $[R_2Fe{-}H]^-$, $[R_2Fe]^-$, $[R_2FeCH_3]^-$, $\{Fe,R_2,O_2\}^-$, $[R_3Fe]^-$
$\{Fe,R_4,O\}^-$	
$[R_2Co]^-$	$[RCoCH_3]^-$, $[R_2Co{-}2CH_4]^-$, $[R_2Co{-}CH_4]^-$
$\{Co,R_2,O_2\}^-$	
$[R_3Co]^-$	$[R_2Co]^-$, $\{Co,R_2,O_2\}^-$
$[R_4Co]^-$	$[R_2Co]^-$, $\{Co,R_2,O_2\}^-$, $[R_3Co]^-$
$[R_5Co_2]^-$	
$[R_2Ni{-}H]^-$	
$[R_2Ni]^-$	$[RNiCH_3]^-$, $[R_2Ni{-}2CH_4]^-$, $[R_2Ni{-}CH_4]^-$, $\{Ni,R_2,O\}^-$, $\{Ni,R_2,O_2\}^-$
$[R_3Ni]^-$	$\{Ni,R,O_2\}^-$, *m/z* = 178.99 (unassigned), $[R_2Ni{-}H]^-$, $[R_2Ni]^-$, $\{Ni,C_6,H_{17},Si_2\}^-$
$[R_4Ni_2{-}H]^-$	
$[R_4Ni]^-$	$[R_2Ni{-}H]^-$, $[R_2Ni]^-$, $[R_3Ni]^-$
$[R_2Cu]^-$	$[R_2CuCH_3]^-$
$[R_3Cu_2]^-$	
$[R_4Cu]^-$	$[R_2Cu]^-$

2.3 CID spectra

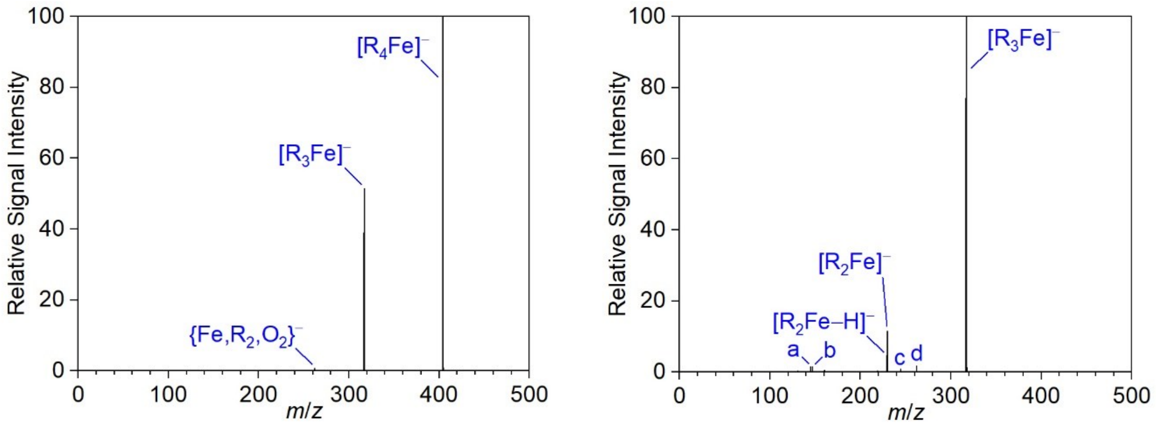

Figure S7. CID spectra of mass-selected $[R_4Fe]^-$ for E_{lab} = 0 eV (left) and 30 eV (right, a = $\{(Fe,R,O,H){-}CH_3\}^-$, b = unassigned, c = $[R_2FeCH_3]^-$, d = $\{Fe,R_2,O_2\}^-$), R = Me_3SiCH_2.

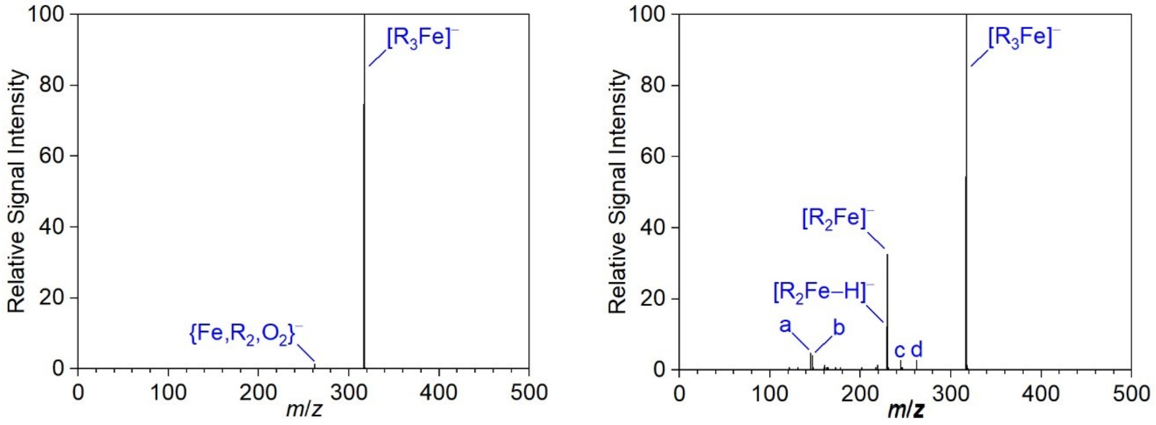

Figure S8. CID spectra of mass-selected $[R_3Fe]^-$ for E_{lab} = 0 eV (left) and 20 eV (right, a = $\{(Fe,R,H,O){-}CH_3\}^-$, b = unassigned, c = $\{R_2FeCH_3\}^-$, d = $\{Fe,R_2,O_2\}^-$), R = Me_3SiCH_2.

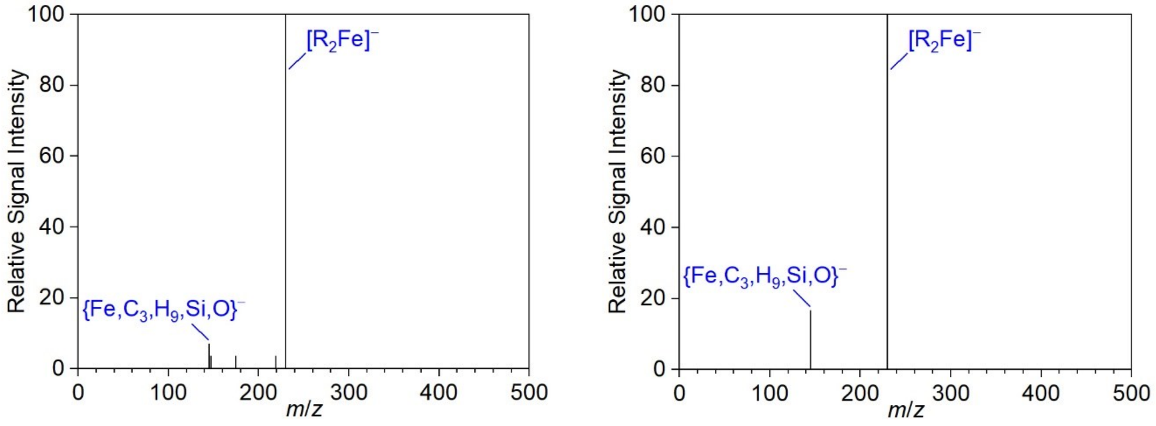

Figure S9. CID spectra of mass-selected $[R_2Fe]^-$ for E_{lab} = 0 eV (left) and 10 eV (right), R = Me_3SiCH_2.

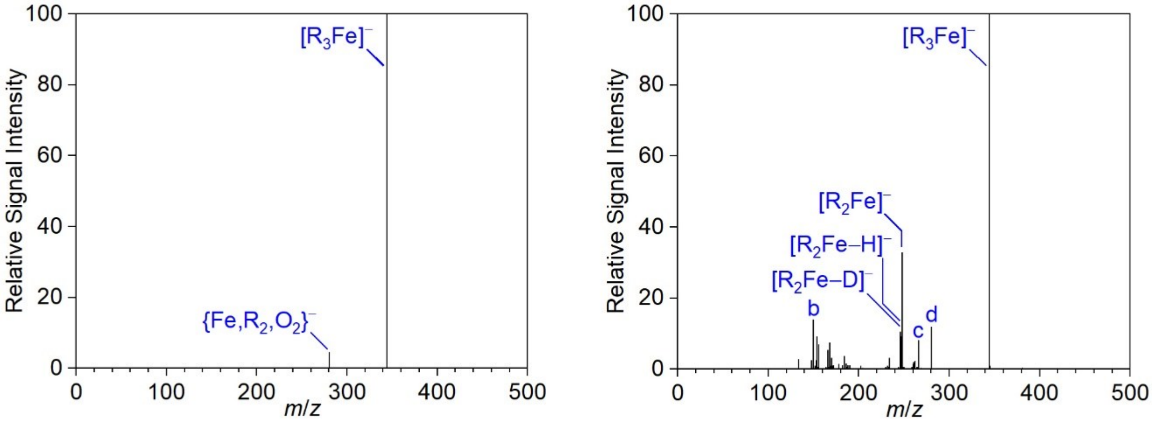

Figure S10. CID spectra of mass-selected $[R_3Fe]^-$ for E_{lab} = 0 eV (left) and 25 eV (right, a = $[RFe{-}CD_3]^-$, b = $\{(Fe,R,O){-}CD_3\}^-$, c = $[R_2FeCD_3]^-$, d = $\{Fe,R_2,O_2\}^-$), R = $(CD_3)_3SiCH_2$.

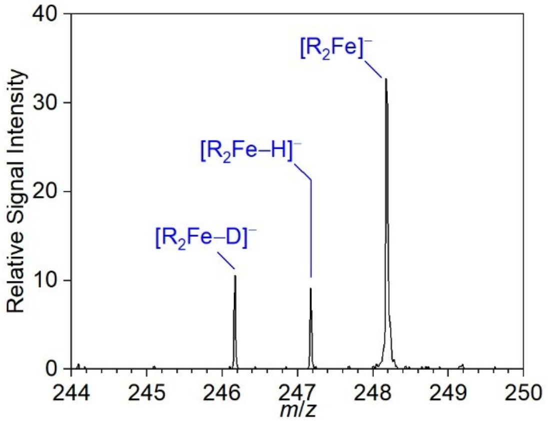

Figure S11. Section of the CID spectrum of mass-selected $[R_3Fe]^-$ for E_{lab} = 25 eV, R = $(CD_3)_3SiCH_2$.

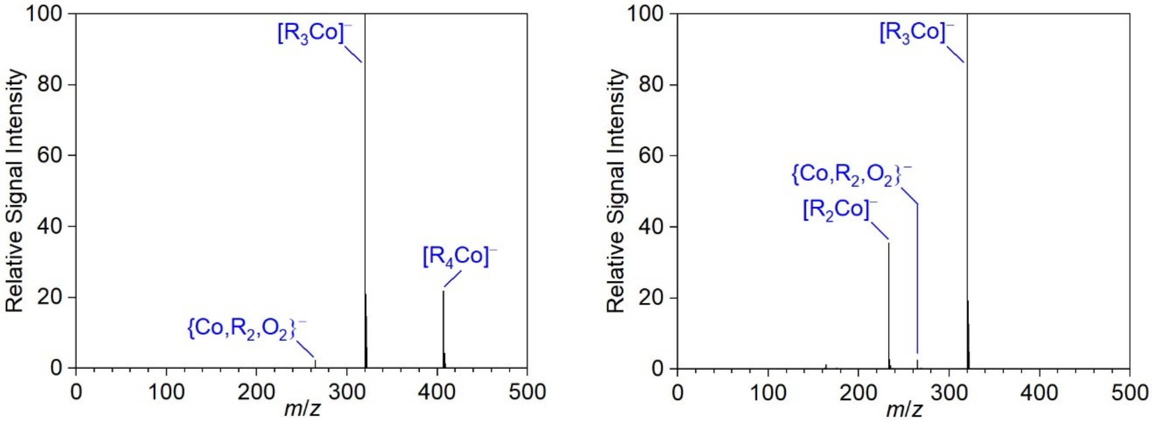

Figure S12. CID spectra of mass-selected $[R_4Co]^-$ for E_{lab} = 0 eV (left) and 15 eV (right), R = Me_3SiCH_2.

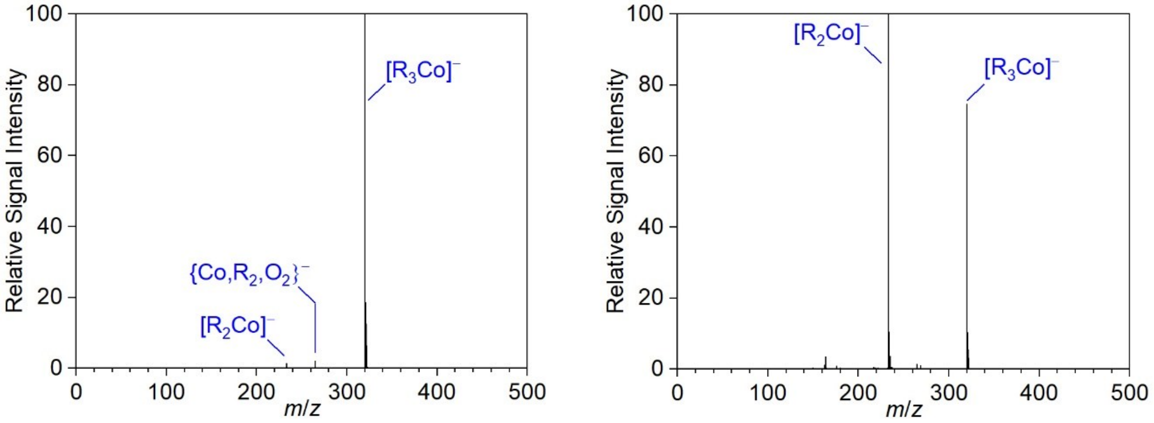

Figure S13. CID spectra of mass-selected $[R_3Co]^-$ for E_{lab} = 0 eV (left) and 8 eV (right), R = Me_3SiCH_2.

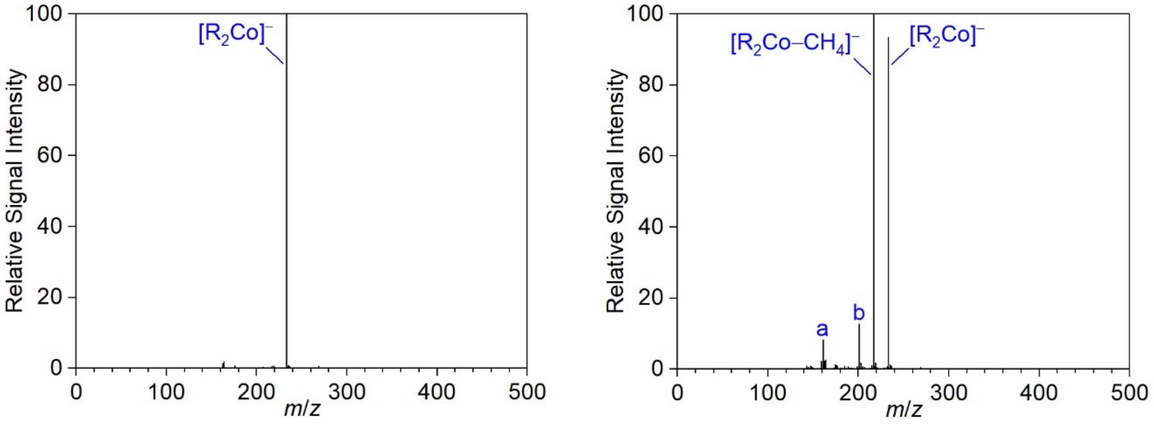

Figure S14. CID spectra of mass-selected $[R_2Co]^-$ for E_{lab} = 0 eV (left) and 13 eV (right, a = $[RCoCH_3]^-$, b = $[R_2Co{-}2CH_4]^-$), R = Me_3SiCH_2.

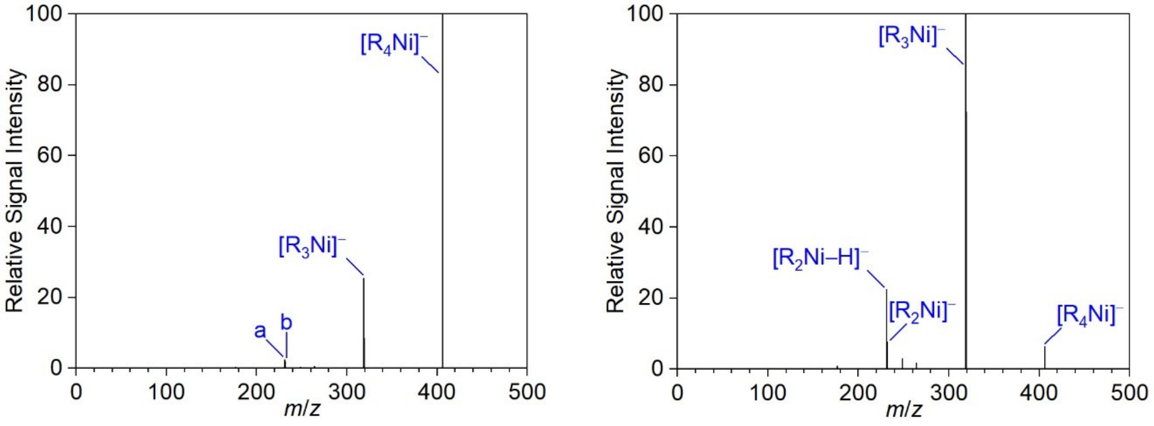

Figure S15. CID spectra of mass-selected $[R_4Ni]^-$ for E_{lab} = 0 eV (left, a = $[R_2Ni{-}H]^-$, b = $[R_2Ni]^-$) and 6 eV (right), R = Me_3SiCH_2.

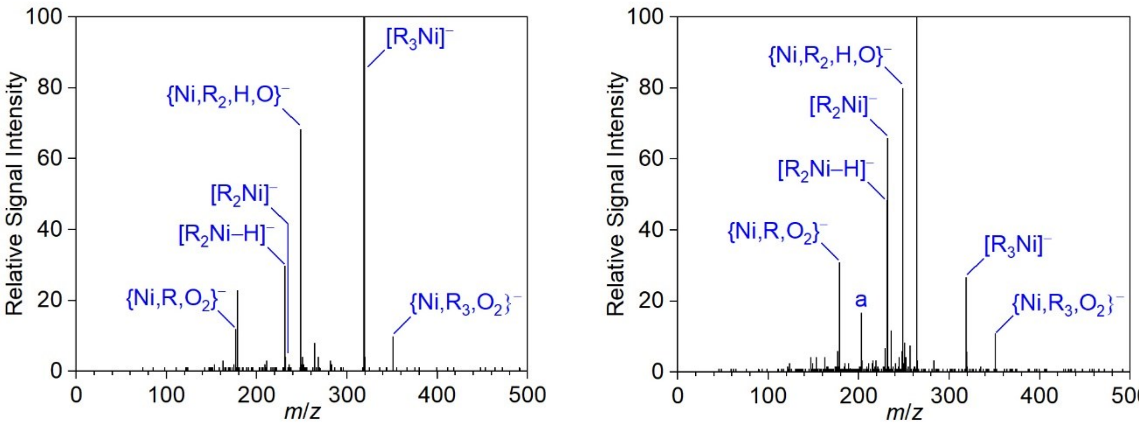

Figure S16. CID spectra of mass-selected $[R_3Ni]^-$ for E_{lab} = 0 eV (left) and 10 eV (right, a = $\{Ni,C_6,H_{17},Si_2\}^-$), R = Me_3SiCH_2.

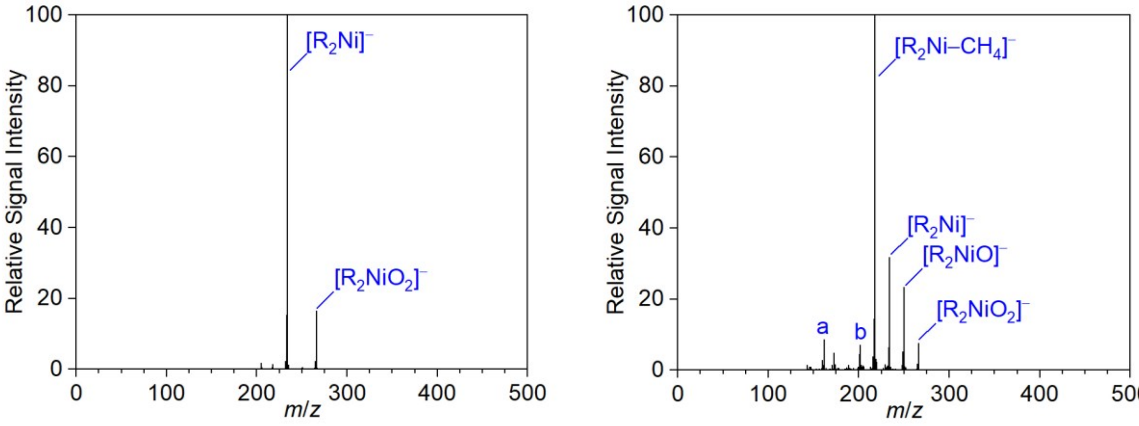

Figure S17. CID spectra of mass-selected $[R_2Ni]^-$ for E_{lab} = 0 eV (left) and 10 eV (right, a = $[RNiCH_3]^-$, b = $[R_2Ni{-}2CH_4]^-$), R = Me_3SiCH_2.

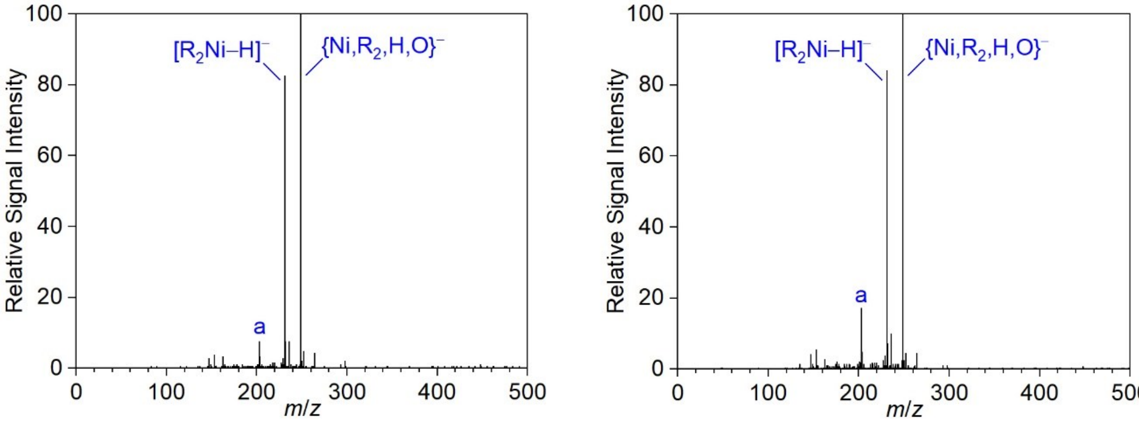

Figure S18. CID spectra of mass-selected $[R_2Ni{-}H]^-$ for E_{lab} = 0 eV (left) and 3 eV (right, a = {Ni,6C,17H,2Si}$^-$), R = Me_3SiCH_2.

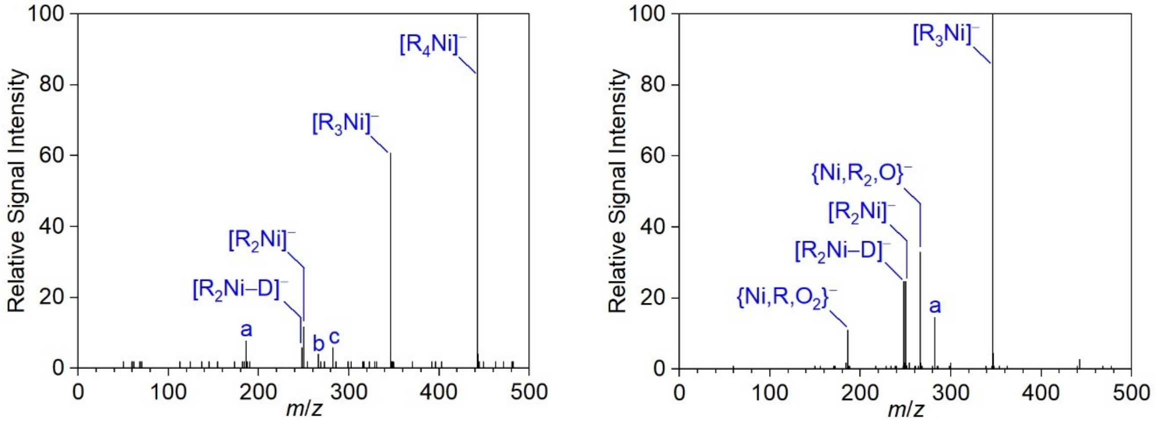

Figure S19. CID spectra of mass-selected $[R_4Ni]^-$ for E_{lab} = 0 eV (left, a = $\{Ni,R,O_2\}^-$, b = $\{Ni,R_2,O\}^-$, c = $\{Ni,R_2,O_2\}^-$) and 9 eV (right, a = $\{Ni,R_2,O_2\}^-$), R = $(CD_3)_3SiCH_2$.

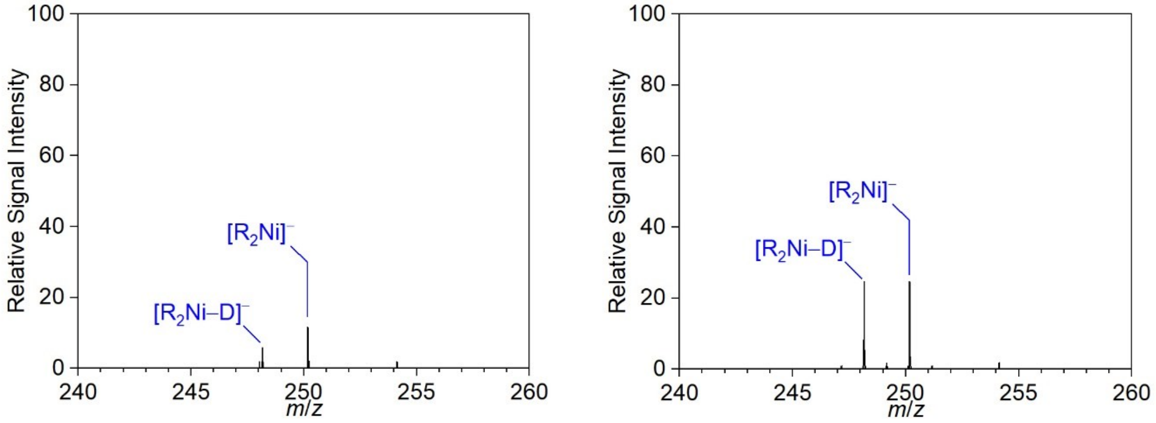

Figure S20. Sections from the CID spectra of mass-selected $[R_4Ni]^-$ for E_{lab} = 0 eV (left) and 9 eV (right), R = $(CD_3)_3SiCH_2$.

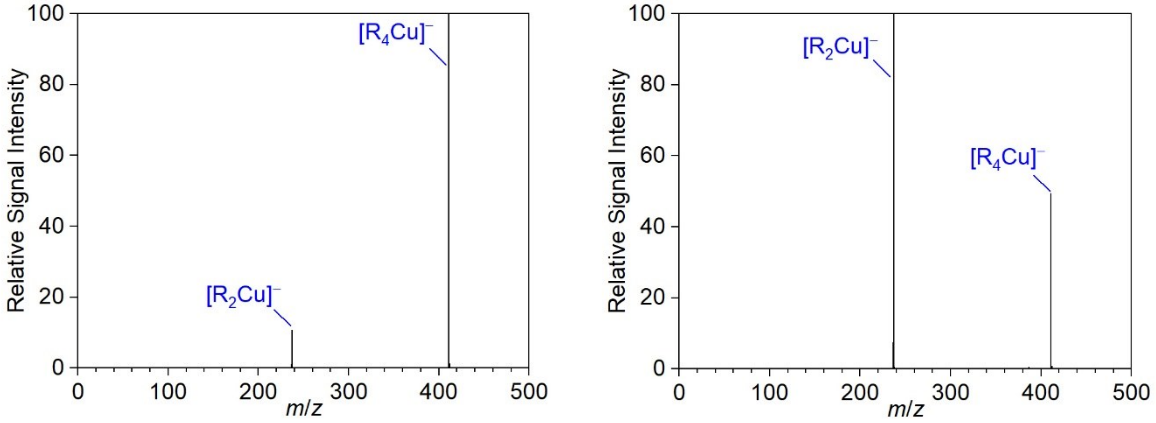

Figure S21. CID spectra of mass-selected $[R_4Cu]^-$ for E_{lab} = 0 eV (left) and 7 eV (right), R = Me_3SiCH_2.

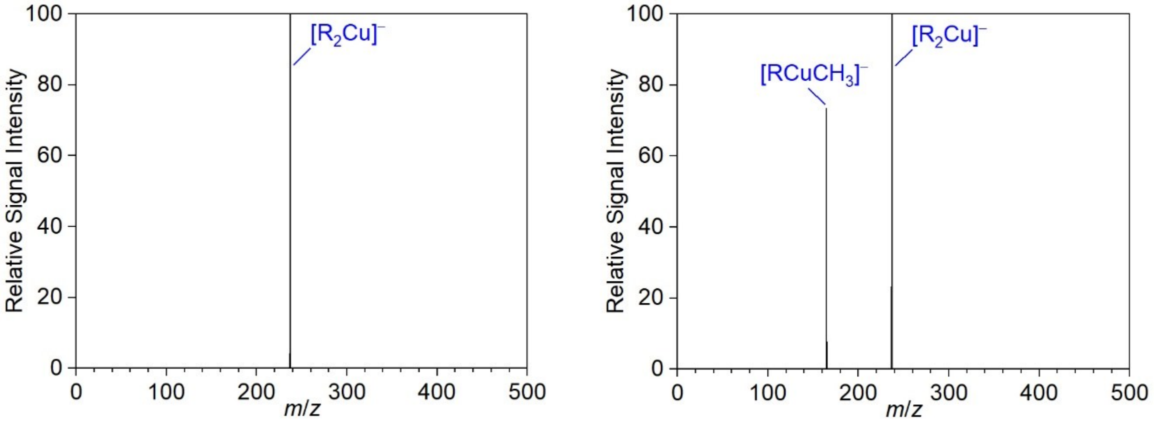

Figure S22. CID spectra of mass-selected $[R_2Cu]^-$ for E_{lab} = 0 eV (left) and 16 eV (right), R = Me_3SiCH_2.

2.4 Additional breakdown curves

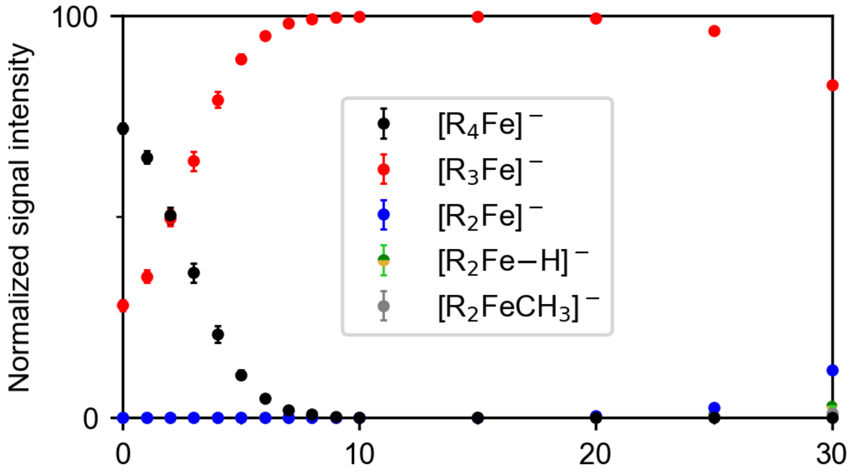

Figure S23. Breakdown curve of $[R_4Fe]^-$, R = Me_3SiCH_2.

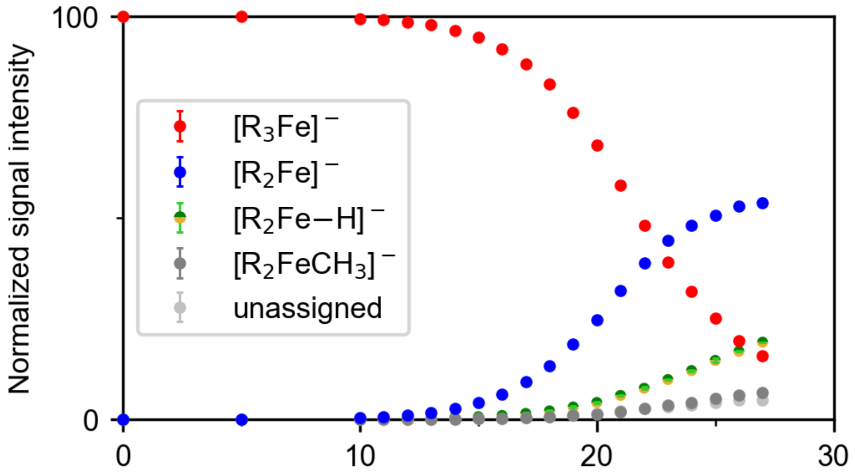

Figure S24. Breakdown curve of $[R_3Fe]^-$, R = Me_3SiCH_2.

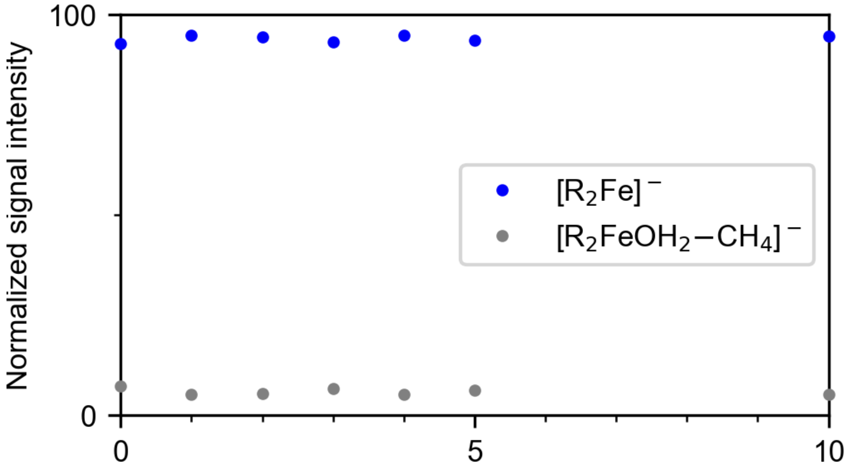

Figure S25. Breakdown curve of $[R_2Fe]^-$, R = Me_3SiCH_2. $[R_2FeOH_2{-}CH_4]^-$ as the only observed product ion is shown here.

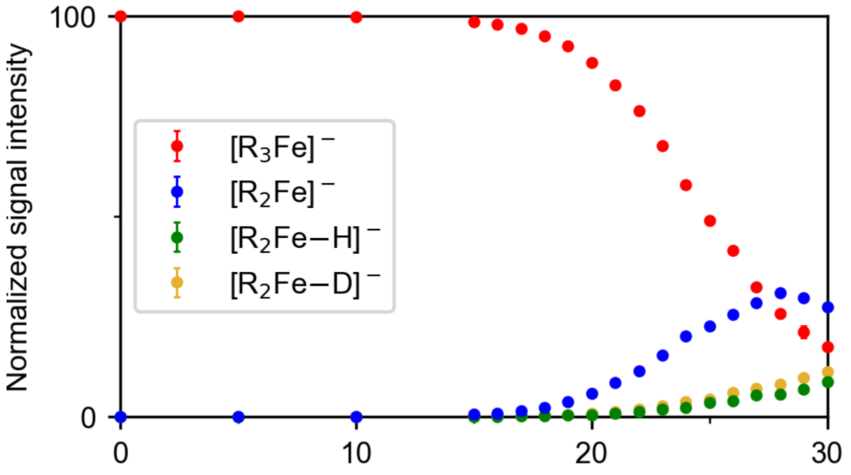

Figure S26. Breakdown curve of $[R_3Fe]^-$, R = $(CD_3)_3SiCH_2$.

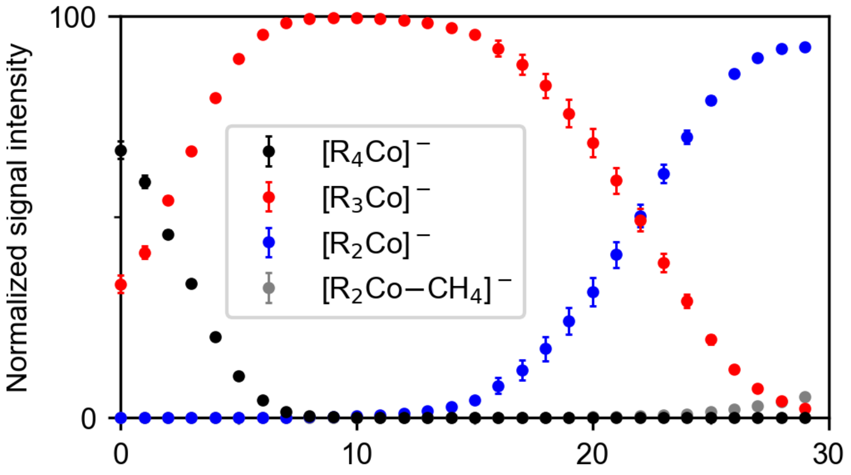

Figure S27. Breakdown curve of $[R_4Co]^-$, R = Me_3SiCH_2.

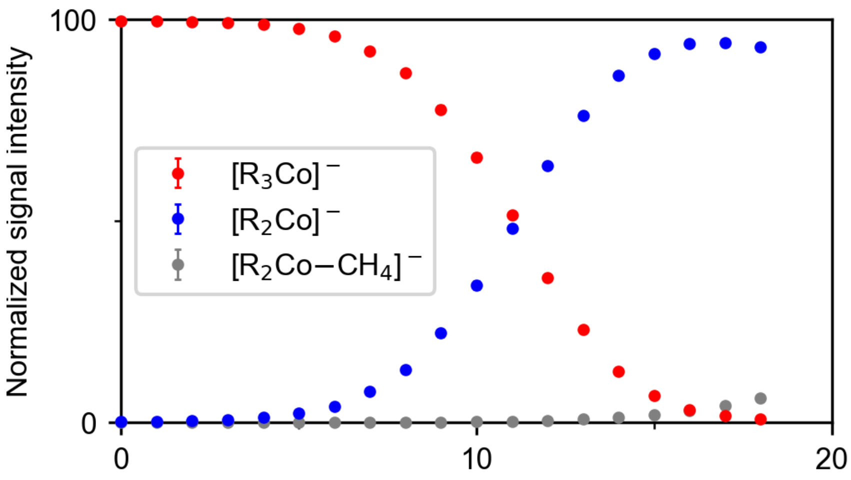

Figure S28. Breakdown curve of $[R_3Co]^-$, R = Me_3SiCH_2.

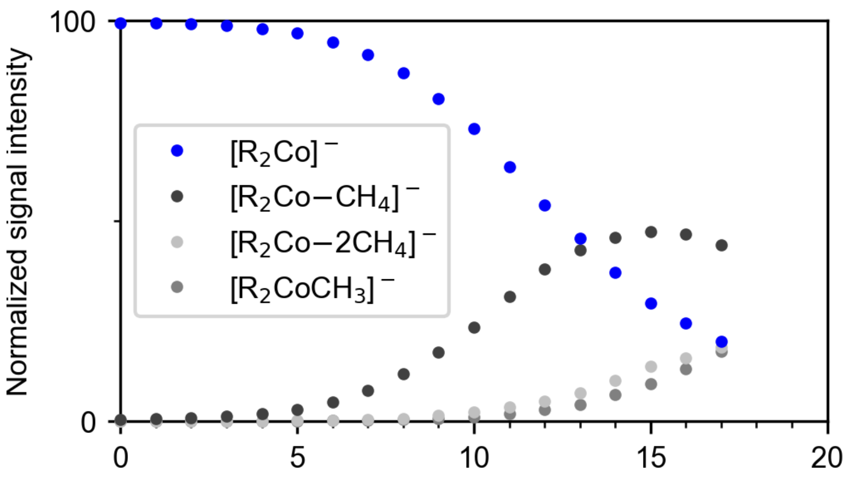

Figure S29. Breakdown curve of $[R_2Co]^-$, R = Me_3SiCH_2.

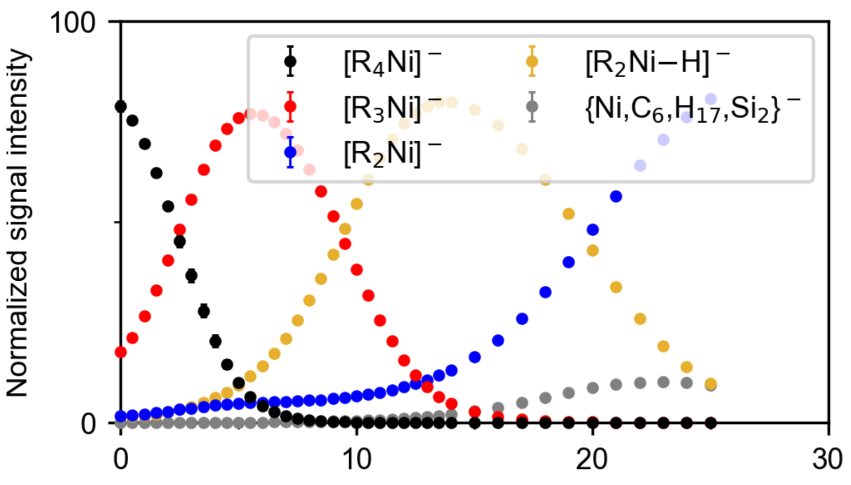

Figure S30. Breakdown curve of $[R_4Ni]^-$, R = Me_3SiCH_2.

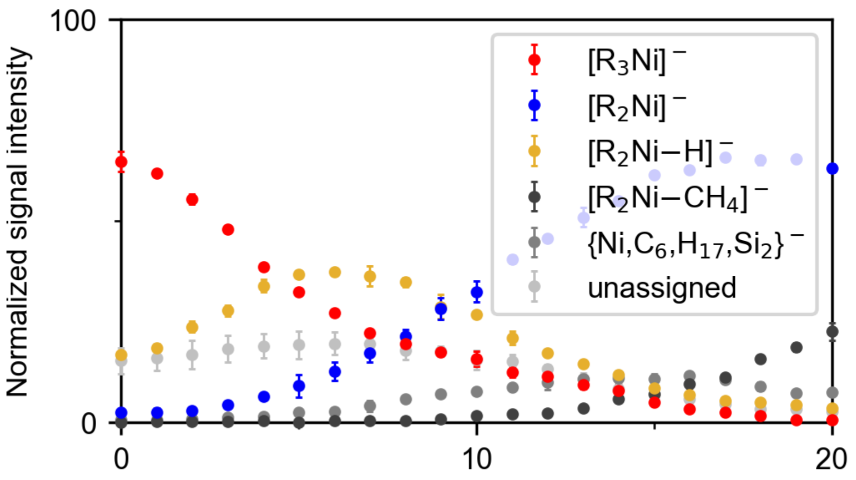

Figure S31. Breakdown curve of $[R_3Ni]^-$, R = Me_3SiCH_2.

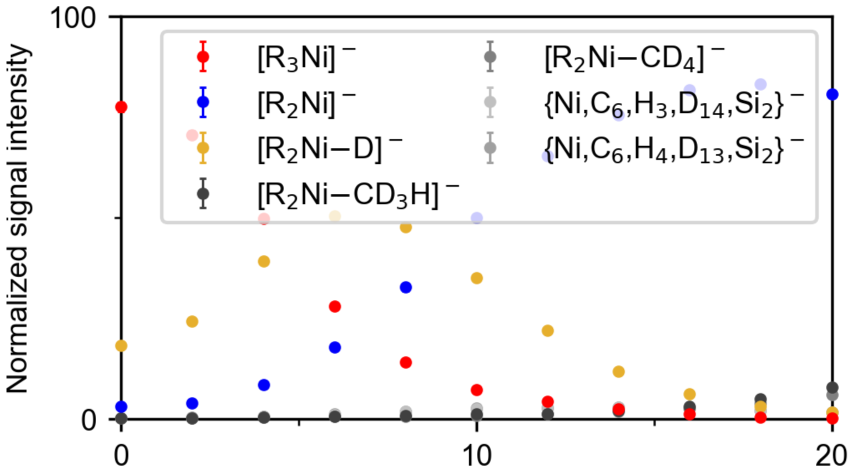

Figure S32. Breakdown curve of $[R_3Ni]^-$, R = $(CD_3)_3SiCH_2$.

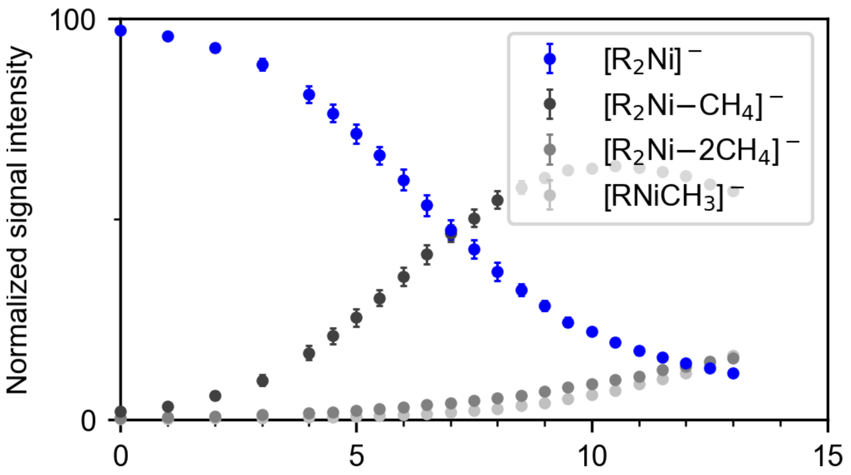

Figure S33. Breakdown curve of $[R_2Ni]^-$, R = Me_3SiCH_2.

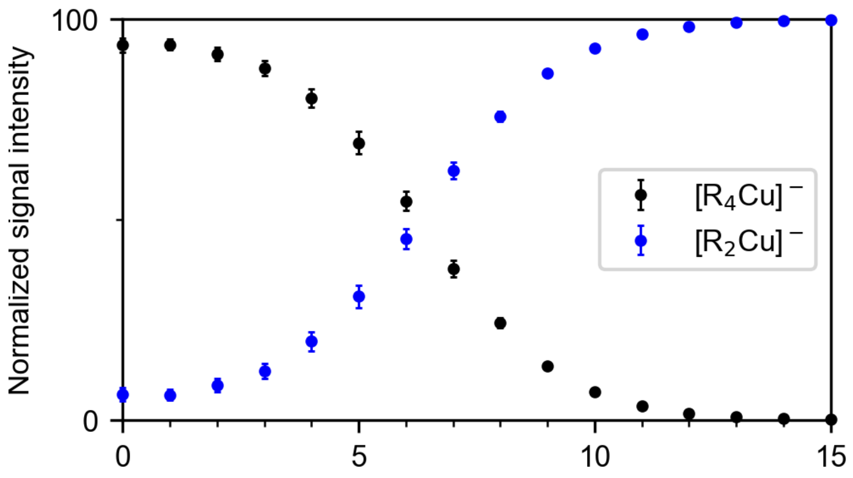

Figure S34. Breakdown curve of $[R_4Cu]^-$, R = Me_3SiCH_2.

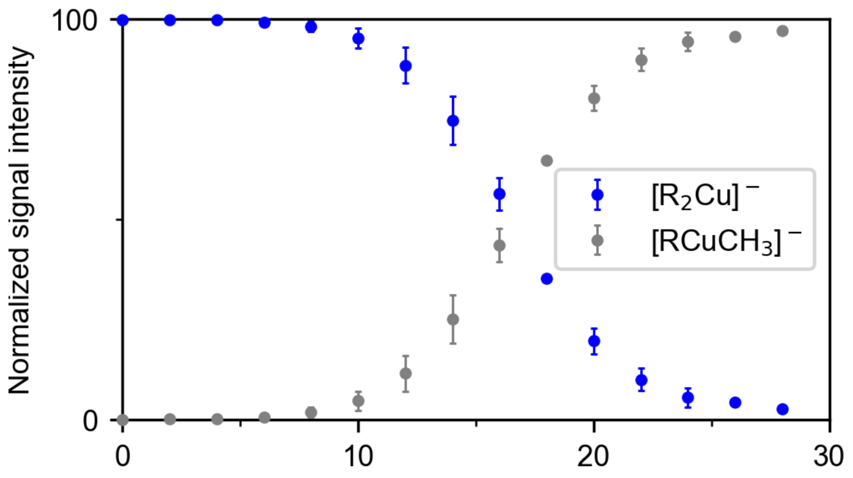

Figure S35. Breakdown curve of $[R_2Cu]^-$, R = Me_3SiCH_2.

3 Computational methods

The molecular structures were optimized using density functional theory (DFT). All DFT calculations were carried out with QChem 6.1.[2] For the complexes containing Fe, Co, and Ni, the ωB97X-D[3] functional was employed in combination with the def2-TZVP[4] basis. The structures of the Cu-centered complexes were optimized using the ωB97M-V[5] functional with the cc-pVTZ[6] basis set and a fully relativistic effective core potential.[7] This choice was made due to the need for a pseudopotential in this case. Previous work demonstrated that the inclusion of exact exchange leads to inaccurate results due to a small amount of static correlation.[8] The final single-point energies were obtained using DLPNO-CCSD(T)[9] in combination with the aug-cc-pVTZ[10] basis set for carbon, silicon, and the respective metals, and the cc-pVTZ[6] basis set for the hydrogen atoms. For the copper-containing complexes, the same effective core potential as for the DFT calculations was employed.[7] The calculations were carried out using Orca version 5.0.3[11] with the *NormalPNO* option, the *VeryTightSCF* option for the self-consistent field procedure and default grid and convergence settings. Zero-point corrections and vibrational frequencies were obtained within the framework of the rigid rotor/harmonic oscillator approximation at standard conditions using the abovementioned DFT methods.

For the statistical-rate theory calculations, the MESS program developed and maintained by Klippenstein and coworkers was employed.[12] Microcanonical rates were computed separately for each unimolecular pathway. Reactions featuring a transition state were treated employing Rice-Ramsperger-Kassel-Marcus (RRKM) theory, neglecting the dependence of the microcanonical rate constants $k(E)$ on the total angular-momentum quantum number J. Again, the rigid rotor/harmonic oscillator approximation was applied to compute the sum of the states of the reactant and the density of states of the transition state utilizing the vibrational frequencies computed via the abovementioned DFT setup. The homolytic bond dissociations of the metal-ligand bond are considered barrierless as it is known from literature for bond fissions of molecular ions.[13] Phase-space theory (PST) was employed to compute the sum of states for the TS in the dissociation limit.[14] For the interaction potential between the radical and the molecular ion, an ion-neutral potential of $-\alpha/r^4$ was employed. The isotropic polarizability α of the radical was computed using the respective DFT methods as 10.75 Å^3.

PST is known to overestimate the rate constants due to the underlying assumption of an isotropic interaction between the dissociated radical and the molecular ion. To account for this shortcoming, the rates were multiplied by an energy-independent rigidity factor of $f_{\mathrm{rigid}} = 10^{-2}$. This value follows recommendations from a previous work, in which the curves $k(E)$ were fitted for the similar propyl-radical loss reaction of the *n*-butylbenzene radical cation to experimental data.[15]

4 Results of electronic structure calculations

4.1 Optimized structures

Optimized structures are saved in XYZ format in units of Å in the file geometries.zip of the Supporting Information. The multiplicity, charge, absolute energy of the DFT calculation, and, if computed, absolute energy of the DLPNO-CCSD(T) and zero-point vibrational energy of the structures are given in the comment line. The vibrational frequencies used in the kinetic calculation are also given for each structure in the corresponding .vibs file.

4.2 Energetics

The energies of the electronic structure calculations for the obtained equilibrium structures and transition states (TS) (see 4.1) are listed for all elements in Tables S4 to S7. The energies and entropic contributions are given relative to the spin ground-state of the $[R_4M]^-$ complex for all elements M=Fe, Co, Ni, Cu.

Table S4. Results of electronic structure calculations for organoferrate ions for each reaction pathway and multiplicity. The reference point is $[R_4Fe]^-$ with a ground-state multiplicity of 4. The difference of the zero-point vibrational energies (ZPVE) ΔZPVE and the entropic contribution $-T\Delta S_{298\text{ K}}$ for 298K to the reference point were calculated with DFT (ωB97X-D/def2-TZVP). The difference of the electronic energy to the reference point is calculated with the same DFT method as above ΔE_{el} (DFT) and additionally with DLPNO-CCSD(T) denoted ΔE_{el} (DLPNO-CCSD(T)). The latter is used for the kinetic calculations. Cells marked with * indicate that the corresponding calculation was not performed as the structure is energetically considerably higher than the ground state (as shown by the initial DFT geometry optimization mentioned above) and is thus considered negligible for the investigated reactivity.

**Structures relative to $[R_4Fe]^-$ (M = 4)**	**Reaction pathway**	**Multiplicity of resulting metalate**	**ΔE_{el} (DFT) (kJ/mol)**	**ΔE_{el} (DLPNO-CCSD(T)) (kJ/mol)**	**ΔZPVE (kJ/mol)**	**$-T\Delta S_{298\text{ K}}$ (kJ/mol)**
$[R_4Fe]^-$	Electronic excitation	6	28.59	21.66	−8.61	−4.34
$[R_3Fe]^-$ + $R^•$	Homolytic dissociation	3	261.85	*	−20.79	−73.33
$[R_3Fe]^-$ + $R^•$	Homolytic dissociation	5	151.34	152.75	−20.31	−70.04
$[R_2Fe]^-$ + 2 $R^•$	Consecutive homolytic dissociations	4	391.60	452.15	−28.16	−123.43
$[R_2Fe]^-$ + 2 $R^•$	Consecutive homolytic dissociations	6	516.29	*	−26.10	−67.66
$[R_2Fe\text{--}R_2]^-$	Reductive elimination (TS)	4	193.71	217.17	−5.33	2.84
$[R_2Fe]^-$ + R_2	Reductive elimination	4	24.25	77.20	−3.40	−71.18

	(coupling product)					
$[R_2Fe{-}{-}R_2]^-$	Reductive elimination (TS)	6	309.95	*	−12.39	−10.44
$[R_2Fe]^-$ + R_2	Reductive elimination (coupling product)	6	148.94	*	−1.34	−67.66
$[R_2{-}HFe{-}{-}RH_\alpha]^-$ + $R^\bullet$	Homolytic dissociation followed by RH_α elimination (TS)	5	388.93	394.76	−32.76	−68.93
$[R_2Fe{-}H]^-$ + $R^\bullet$ + RH_α	Homolytic dissociation followed by RH_α elimination (products)	5	345.38	349.34	−22.96	−126.61
$[R_2{-}HFe{-}{-}RH_\gamma]^-$ + $R^\bullet$	Homolytic dissociation followed by insertion/elimination (TS)	5	346.18	348.64	−31.12	−60.46
$[R_2Fe{-}H]^-$ + $R^\bullet$+RH_γ	Homolytic dissociation followed by insertion/elimination (products)	5	253.93	255.72	−25.40	−118.46

Table S5. Results of electronic structure calculations for cobaltate ions for each reaction pathway and multiplicity. The reference point is the $[R_4Co]^-$ with its ground-state multiplicity of 3. The difference of the zero-point vibrational energies (ZPVE) ΔZPVE and the entropic contribution $-T\Delta S_{298\,K}$ for 298K to the reference point were calculated with DFT (ωB97X-D/def2-TZVP). The difference of the electronic energy to the reference point is calculated with the same DFT method as above ΔE_{el} (DFT) and additionally with DLPNO-CCSD(T) denoted ΔE_{el} (DLPNO-CCSD(T)). The latter data is used for the kinetic calculations.

**Structures relative to $[R_4Co]^-$ (M = 3)**	**Reaction pathway**	**Multiplicity of resulting metalate**	**ΔE_{el} (DFT) (kJ/mol)**	**ΔE_{el} (DLPNO-CCSD(T)) (kJ/mol)**	**ΔZPVE (kJ/mol)**	**$-T\Delta S_{298\,K}$ (kJ/mol)**
$[R_3Co]^- + R^\bullet$	Homolytic dissociation	2	217.75	242.94	−18.95	−65.00
$[R_3Co]^- + R^\bullet$	Homolytic dissociation	4	158.45	137.40	−20.18	−67.43
$[R_2Co]^- + 2\ R^\bullet$	Consecutives homolytic dissociations	3	346.01	363.20	−26.61	−121.34
$[R_2Co]^- + 2\ R^\bullet$	Consecutive homolytic dissociations	5	522.99	478.38	−26.12	−117.01
$[R_2Co\text{--}R_2]^-$	Reductive elimination (TS)	3	164.58	152.42	−4.26	10.43
$[R_2Co]^- + R_2$	Reductive elimination (coupling product)	3	−21.34	−11.76	−1.84	−69.10
$[R_2\text{–}HCo\text{--}RH_\alpha]^- + R^\bullet$	Homolytic dissociation followed by RH_α elimination (TS)	4	366.42	368.05	−32.43	−65.22
$[R_2Co\text{–}H]^- + R^\bullet + RH_\alpha$	Homolytic dissociation followed by RH_α elimination (products)	4	351.98	317.20	−22.84	−114.03
$[R_2\text{–}HCo\text{--}RH_\gamma]^- + R^\bullet$	Homolytic dissociation followed by insertion (TS)	4	341.38	319.60	−31.49	−60.07
$[R_2Co\text{–}H]^- + R^\bullet + RH_\gamma$	Homolytic dissociation followed by insertion (products)	4	250.54	236.25	−23.99	−116.00

Table S6. Results of electronic structure calculations for Ni species for each reaction pathway and multiplicity. The reference point is the $[R_4Ni]^-$ with its ground-state multiplicity of 2. The difference of the zero-point vibrational energies (ZPVE) ΔZPVE and the entropic contribution $-T\Delta S_{298\,K}$ for 298K to the reference point were calculated with DFT (ωB97X-D/def2-TZVP). The difference of the electronic energy to the reference point is calculated with the same DFT method as above ΔE_{el} (DFT) and additionally with DLPNO-CCSD(T) denoted ΔE_{el} (DLPNO-CCSD(T)). The latter data is used for the kinetic calculations.

**Structures relative to $[R_4Ni]^-$ (M = 2)**	**Reaction pathway**	**Multiplicity of resulting metalate**	**ΔE_{el} (DFT) (kJ/mol)**	**ΔE_{el} (DLPNO-CCSD(T)) (kJ/mol)**	**ΔZPVE (kJ/mol)**	**$-T\Delta S_{298\,K}$ (kJ/mol)**
$[R_3Ni]^- + R^•$	Homolytic dissociation	1	168.55	205.13	−15.43	−60.49
$[R_3Ni]^- + R^•$	Homolytic dissociation	3	160.57	202.51	−17.14	−62.64
$[R_2Ni]^- + 2\,R^•$	Consecutive homolytic dissociations	2	348.61	390.34	−24.92	−112.76
$[R_2Ni\text{--}R_2]^-$	Reductive elimination (TS)	2	123.59	152.79	−4.57	4.70
$[R_2Ni]^- + R_2$	Reductive elimination (coupling product)	2	−18.74	15.38	−0.15	−122.42
$[R_2\text{–}HNi\text{--}RH_\alpha]^- + R^•$	Homolytic dissociation followed by RH_α eliminat-ion (TS)	1	374.41	402.63	−23.95	−55.13
$[R_2Ni\text{–}H]^- + R^• + RH_\alpha$	Homolytic dissociation followed by RH_α eliminat-ion (products)	1	321.40	369.08	−19.00	−112.16
$[R_2\text{–}HNi\text{--}RH_\gamma]^- + R^•$	Homolytic dissociation followed by insertion (TS)	1	263.48	293.31	−23.75	−52.73
$[R_2Ni\text{–}H]^- + R^• + RH_\gamma$	Homolytic dissociation followed by insertion (products)	1	238.35[a]	69.75[a]	−21.22	−112.16
$[R_2\text{–}HNi\text{--}RH_\alpha]^- + R^•$	Homolytic dissociation followed by RH_α elimination (TS)	3	355.69	434.68	−32.23	−62.31
$[R_2Ni\text{–}H]^- + R^• + RH_\alpha$	Homolytic dissociation	3	310.04	371.82	−23.69	−120.04

	followed by RH_α elimination (products)					
$[R_2{-}HNi{-}{-}RH_\gamma]^-$ + $R^\bullet$	Homolytic dissociation followed by insertion (TS)	3	342.44	382.18	−29.45	−55.18
$[R_2Ni{-}H]^-$ + $R^\bullet$ + RH_γ	Homolytic dissociation followed by insertion (products)	3	251.33	295.73	−24.10	−112.33

[a] The large discrepancy between the DFT and DLPNO-CCSD(T) results is surprising and unique to this particular pathway. No convergence problems or similar technical issues were observed. Since the kinetic analysis is not affected (we do not observe a similar discrepancy for the transition-state energy), no conclusion drawn in the main manuscript is affected by this oddity.

Table S7. Results of electronic structure calculations for Cu species for each reaction pathway and multiplicity. The reference point is the $[R_4Cu]^-$ with its ground-state multiplicity of 1. The difference of the zero-point vibrational energies (ZPVE) ΔZPVE and the entropic contribution $-T\Delta S_{298\text{ K}}$ for 298K to the reference point were calculated with DFT (ωB97M-V/cc-pVTZ). The difference of the electronic energy to the reference point is calculated with the same DFT method as above ΔE_{el} (DFT) and additionally with DLPNO-CCSD(T) denoted ΔE_{el} (DLPNO-CCSD(T)). The latter data is used for the kinetic calculations.

**Structures relative to $[R_4Cu]^-$ (M = 1)**	**Reaction pathway**	**Multiplicity of resulting metalate**	**ΔE_{el} (DFT) (kJ/mol)**	**ΔE_{el} (DLPNO-CCSD(T)) (kJ/mol)**	**ΔZPVE (kJ/mol)**	**$-T\Delta S_{298\text{ K}}$ (kJ/mol)**
$[R_3Cu]^- + R^\bullet$	Homolytic dissociation	2	203.51	246.69	−24.89	−45.39
$[R_2Cu]^- + 2\ R^\bullet$	Consecutive homolytic dissociations	1	303.03	340.23	−32.97	−130.78
$[R_2Cu\text{--}R_2]^-$	Reductive elimination (TS)	2	140.88	147.44	−7.91	−3.81
$[R_2Cu]^- + R_2$	Reductive elimination (coupling product)	2	−73.35	−69.72	−7.49	−74.67

[1] G. Thomas, S. Donnecke, I. C. Chagunda, J. S. McIndoe, *Chem. Meth*. **2022**, *2*, e202100101.

[2] E. Epifanovsky, A. T. B. Gilbert, X. Feng, J. Lee, Y. Mao, N. Mardirossian, P. Pokhilko, A. F. White, M. P. Coons, A. L. Dempwolff, Z. Gan, D. Hait, P. R. Horn, L. D. Jacobson, I. Kaliman, J. Kussmann, A. W. Lange, K. U. Lao, D. S. Levine, J. Liu, S. C. McKenzie, A. F. Morrison, K. D. Nanda, F. Plasser, D. R. Rehn, M. L. Vidal, Z.-Q. You, Y. Zhu, B. Alam, B. J. Albrecht, A. Aldossary, E. Alguire, J. H. Andersen, V. Athavale, D. Barton, K. Begam, A. Behn, N. Bellonzi, Y. A. Bernard, E. J. Berquist, H. G. A. Burton, A. Carreras, K. Carter-Fenk, R. Chakraborty, A. D. Chien, K. D. Closser, V. Cofer-Shabica, S. Dasgupta, M. de Wergifosse, J. Deng, M. Diedenhofen, H. Do, S. Ehlert, P.-T. Fang, S. Fatehi, Q. Feng, T. Friedhoff, J. Gayvert, Q. Ge, G. Gidofalvi, M. Goldey, J. Gomes, C. E. González-Espinoza, S. Gulania, A. O. Gunina, M. W. D. Hanson-Heine, P. H. P. Harbach, A. Hauser, M. F. Herbst, M. Hernández Vera, M. Hodecker, Z. C. Holden, S. Houck, X. Huang, K. Hui, B. C. Huynh, M. Ivanov, Á. Jász, H. Ji, H. Jiang, B. Kaduk, S. Kähler, K. Khistyaev, J. Kim, G. Kis, P. Klunzinger, Z. Koczor-Benda, J. H. Koh, D. Kosenkov, L. Koulias, T. Kowalczyk, C. M. Krauter, K. Kue, A. Kunitsa, T. Kus, I. Ladjánszki, A. Landau, K. V. Lawler, D. Lefrancois, S. Lehtola, R. R. Li, Y.-P. Li, J. Liang, M. Liebenthal, H.-H. Lin, Y.-S. Lin, F. Liu, K.-Y. Liu, M. Loipersberger, A. Luenser, A. Manjanath, P. Manohar, E. Mansoor, S. F. Manzer, S.-P. Mao, A. V. Marenich, T. Markovich, S. Mason, S. A. Maurer, P. F. McLaughlin, M. F. S. J. Menger, J.-M. Mewes, S. A. Mewes, P. Morgante, J. W. Mullinax, K. J. Oosterbaan, G. Paran, A. C. Paul, S. K. Paul, F. Pavošević, Z. Pei, S. Prager, E. I. Proynov, Á. Rák, E. Ramos-Cordoba, B. Rana, A. E. Rask, A. Rettig, R. M. Richard, F. Rob, E. Rossomme, T. Scheele, M. Scheurer, M. Schneider, N. Sergueev, S. M. Sharada, W. Skomorowski, D. W. Small, C. J. Stein, Y.-C. Su, E. J. Sundstrom, Z. Tao, J. Thirman, G. J. Tornai, T. Tsuchimochi, N. M. Tubman, S. P. Veccham, O. Vydrov, J. Wenzel, J. Witte, A. Yamada, K. Yao, S. Yeganeh, S. R. Yost, A. Zech, I. Y. Zhang, X. Zhang, Y. Zhang, D. Zuev, A. Aspuru-Guzik, A. T. Bell, N. A. Besley, K. B. Bravaya, B. R. Brooks, D. Casanova, J.-D. Chai, S. Coriani, C. J. Cramer, G. Cserey, A. E. DePrince, R. A. DiStasio, A. Dreuw, B. D. Dunietz, T. R. Furlani, W. A. Goddard, S. Hammes-Schiffer, T. Head-Gordon, W. J. Hehre, C.-P. Hsu, T.-C. Jagau, Y. Jung, A. Klamt, J. Kong, D. S. Lambrecht, W. Liang, N. J. Mayhall, C. W. McCurdy, J. B. Neaton, C. Ochsenfeld, J. A. Parkhill, R. Peverati, V. A. Rassolov, Y. Shao, L. V. Slipchenko, T. Stauch, R. P. Steele, J. E. Subotnik, A. J. W. Thom, A. Tkatchenko, D. G. Truhlar, T. Van Voorhis, T. A. Wesolowski, K. B. Whaley, H. L. Woodcock, P. M. Zimmerman, S. Faraji, P. M. W. Gill, M. Head-Gordon, J. M. Herbert, A. I. Krylov, *J. Chem. Phys.* **2021**, *155*, 084801.

[3] a) J.-D. Chai, M. Head-Gordon, *J. Chem. Phys*. **2008**, *128*, 084106, b) S. Grimme, J. Antony, S. Ehrlich, H. Krieg, *J. Chem. Phys.* **2010**, *132*, 154104.

[4] a) F. Weigend, F. Furche, R. Ahlrichs, *J. Chem. Phys.* **2003**, *119*, 12753–12762, b) F. Weigend, R. Ahlrichs, *Phys. Chem. Chem. Phys.* **2005**, *7*, 3297–3305.

[5] N. Mardirossian, M. Head-Gordon, *J. Chem. Phys.* **2016**, *144*, 214110.

[6] T. H. Dunning, *J. Chem. Phys.* **1989**, *90*, 1007–1023.
[7] D. Figgen, G. Rauhut, M. Dolg, H. Stoll, *Chem. Phys.* **2005**, *311*, 227–244.
[8] T. Auth, C. Stein, R. A. J. O'Hair, K. Koszinowski, *Chem. Eur. J.* **2021**, *28*, e202103130.
[9] a) C. Riplinger, F. Neese, *J. Chem. Phys.* **2013**, *138*, 034106, b) C. Riplinger, B. Sandhoefer, A. Hansen, F. Neese, *J. Chem. Phys.* **2013**, *139*, 134101, c) C. Riplinger, P. Pinski, U. Becker, E. F. Valeev, F. Neese, *J. Chem. Phys.* **2016**, *144*, 024109.
[10] D. E. Woon, T. H. Dunning, *J. Chem. Phys.* **1993**, *98*, 1358–1371.
[11] F. Neese, *WIREs Comput. Mol. Sci*. **2022**, *12*, e1606.
[12] a) Y. Georgievskii, C. F. Goldsmith, J. A. Miller, M. P. Burke, S. J. Klippenstein, MESS.2016.3.23, Master Equation System Solver, **2016**, b) Y. Georgievskii, J. A. Miller, M. P. Burke, S. J. Klippenstein, *J. Phys. Chem. A*, **2013**, *117*, 12146–12154.
[13] P. B. Armentrout, K. M. Ervin, M. T. Rodgers, *J. Phys. Chem. A* **2008**, *112*, 10071–10085.
[14] J. C. Light, *J. Chem. Phys.* **1964**, *40*, 3221–3229.
[15] J. Troe, *J. Chem. Soc. Faraday Trans.* **1997**, *93*, 885–891, b) S. Halbert, G. Bouchoux, *J. Phys. Chem. A* **2012**, *116*, 1307–1315.
